# Acute hyperexcitability differentially affects hippocampal neurogenesis features and spatial memory

**DOI:** 10.3389/fncel.2026.1833859

**Published:** 2026-06-02

**Authors:** Diana López-Ibarra, Andrea Aguilar-Arredondo, Verónica Gaytan-Zeron, Karina Hernández-Mercado, Teresa Montiel, Daniel Osorio-Gómez, Angélica Zepeda

**Affiliations:** 1 Instituto de Investigaciones Biomédicas, Universidad Nacional Autónoma de México, UNAM, Mexico City, Mexico; 2 Laboratorio de Plasticidad Neuronal Leloir Institute (IIBBA-CONICET), Buenos Aires, Argentina; 3 Instituto de Fisiología Celular, Universidad Nacional Autónoma de México, UNAM, Mexico City, Mexico; 4 Laboratorio de Algoritmos y Fisiología del Cerebro, Leloir Institute (IIBBA-CONICET), Buenos Aires, Argentina

**Keywords:** acute seizures, adult hippocampal neurogenesis, adult hippocampus, behavior, brain damage, contextual fear conditioning and memory (CFC), dentate gyrus, brain plasticity

## Abstract

**Introduction:**

Epileptic seizures induce aberrant adult hippocampal neurogenesis (AHN), yet how varying seizure intensities affect this process and associated memory functions remains unclear.

**Methods:**

Here we administered systemically a low (5 mg/kg) or a high (25 mg/kg) dose of kainic acid (KA) to induce acute hyperexcitability of differing severity in mice of both sexes (6 to 8 weeks-old) genetically labeled for Ascl1, which allowed to follow progenitor cells and their progeny in the dentate gyrus (DG). We recorded EEG, assessed spatial and contextual fear memory, and analyzed adult-born granule cells (abGCs) 32 days post-treatment.

**Results:**

High-dose KA produced more severe and prolonged seizures, increased mature abGCs with altered laminar positioning, and elevated dendritic spine density, whereas low-dose KA induced presynaptic bouton enlargement. Both doses impaired spatial location recognition but spared contextual fear memory.

**Discussion:**

These findings reveal that acute seizure severity differentially modulates morphological and synaptic features of abGCs and selectively disrupts hippocampal-dependent spatial memory, indicating that hyperexcitability exerts qualitative effects on neurogenesis and cognitive function.

## Introduction

Adult hippocampal neurogenesis (AHN) is a form of neural plasticity that persists throughout life (Altman and Das, 1965) and plays a fundamental role in hippocampal-dependent learning and memory (Terranova et al., 2019; Chavan et al., 2025). In mammals, adult neurogenesis is restricted to two brain regions: the subventricular zone (SVZ), which lines the lateral ventricles, and the subgranular zone (SGZ) of the dentate gyrus (DG), where hippocampal neurogenesis occurs (Altman and Das, 1965; Kempermann et al., 2015; Lois and Alvarez-Buylla, 1993).

In the DG, adult-born granule cells (abGCs) originate from neural progenitor cells, migrate into the granule cell layer (GCL), and progressively integrate into existing hippocampal circuits (for reviews see: Denoth-Lippuner and Jessberger, 2021; Kempermann et al., 2015; Zhao et al., 2006). Proper migration and positioning of abGCs are essential for their functional integration into hippocampal circuits. This process depends on extrinsic and intrinsic signaling mechanisms (Faigle and Song, 2013).

Intrinsic signaling cascades involving chemotactic molecules such as netrins and semaphorins, as well as extracellular matrix proteins such as reelin, play a key role in neuronal migration and organization of the DG (Ayala et al., 2007). Reelin, secreted by Cajal–Retzius cells and hilar interneurons, is a critical regulator of granule cell positioning, and alterations in these cellular populations can disrupt the migration and positioning of adult-born neurons (Del Río et al., 1997; Santana and Marzolo, 2017).

Adult-born neurons initially exhibit an immature phenotype characterized by short processes, rounded morphology, and radial migration toward the GCL. During maturation, these neurons extend dendrites through the GCL toward the molecular layer and project axons to CA3 (Espósito et al., 2005; Zhao et al., 2006). Although a substantial proportion of immature neurons undergo apoptosis, those that survive acquire a mature granule cell phenotype approximately 8 weeks after differentiation (Overstreet-Wadiche et al., 2006; Song et al., 2012; Van Praag et al., 2002). AHN is highly sensitive to changes in the neurogenic niche, defined by the cellular and molecular environment of the SGZ. Several conditions negatively modulate neurogenesis, including drugs, stress, aging, and neurodegenerative diseases (Boldrini et al., 2018; Morris et al., 2010; Snyder et al., 2011; Tobin et al., 2019). On the other hand, physical exercise and environmental enrichment enhance neurogenesis by increasing progenitor proliferation and neuronal survival, respectively (Olson et al., 2006). Notably, pathological conditions such as ischemic injury, excitotoxic lesions, and epileptic seizures have also been shown to stimulate neurogenesis (Aguilar-Arredondo and Zepeda, 2018; Parent et al., 1997; Tang et al., 2022; Zepeda et al., 2013).

Epilepsy is a group of neurological disorders characterized by recurrent epileptic seizures and associated functional impairments (Devinsky et al., 2018). At the cellular level, seizures are defined by neuronal hyperexcitability and hypersynchronization, resulting from an imbalance between excitatory glutamatergic transmission and inhibitory GABAergic signaling (Fisher et al., 2005; Kourdougli et al., 2017). Epileptic seizures induce structural and functional alterations in the hippocampus, with the DG being particularly susceptible to excitotoxic damage (Ben-Ari et al., 1980). Kainic acid (KA)-induced seizures produce a well-characterized pattern of hippocampal pathology, including neuronal loss, mossy fiber sprouting, reactive gliosis, and blood–brain barrier dysfunction, but also subtle changes at the ultrastructural level (Zhvania et al., 2025). The dentate gyrus is particularly vulnerable to excitotoxic injury, and granule cell dispersion is especially prominent when KA is administered intracerebrally (Blümcke et al., 2012; Buckmaster, 2012; Hernández-Mercado et al., 2022; Ortinski et al., 2010). These structural alterations have been associated with functional impairments in hippocampal-dependent learning and memory, particularly in tasks involving spatial memory and contextual processing (Clelland et al., 2009; Inostroza et al., 2011).

Regarding neurogenesis studies, experimental models of status epilepticus (SE) have shown increased proliferation of neural progenitor cells as well as an abnormal migration of abGCs (Jessberger et al., 2005; Kron et al., 2010; Lybrand et al., 2021; Parent and Murphy, 2008).

Seizure-induced neurogenesis has been associated with altered neuronal excitability and synaptic integration, suggesting that adult-born neurons may influence hippocampal network dynamics in the epileptic brain (Jakubs et al., 2006; Overstreet-Wadiche and Westbrook, 2006; Van Praag et al., 2002). Despite accelerated maturation and functional integration, seizure-induced abGCs often exhibit aberrant morphology and migration (Jessberger et al., 2005; Jessberger and Parent, 2015; Parent, 2007; Parent et al., 1997). These alterations include the extension of basal dendrites toward the hilus, ectopic localization within the hilus or CA3 border, and the presence of immature dendritic spines (Scharfman et al., 2000; Shapiro and Ribak, 2006). Also, several studies suggest that a proportion of these neurons successfully integrate into dentate gyrus circuits, contributing to changes in network connectivity (Kron et al., 2010; Lybrand et al., 2021). The functional contribution of seizure-induced neurogenesis remains controversial, as both pro-epileptogenic and neuroprotective roles have been proposed (Cho and Hsieh, 2024; Iyengar et al., 2025; Jain et al., 2019; Jung et al., 2006). Interestingly, the impact of epileptic activity on adult hippocampal neurogenesis is not uniform and depends on seizure severity. While severe seizures and chronic epilepsy are associated with long-term depletion of neural progenitors and impaired neuronal survival, milder or acute epileptic events may differentially affect distinct phases of the neurogenic process (Mohapel et al., 2004; Yang et al., 2008).

Previous work has demonstrated that different levels of hyperexcitability, as well as different chemoconvulsants, selectively modulate progenitor activation, proliferation, and lineage commitment (Moura et al., 2020; Sierra et al., 2015). However, the effects of these factors on neuronal maturation and the development of synaptic structures, which may influence the integration of new neurons, remain to be explored.

In this context, the present study aims to investigate how different levels of epileptic activity shape AHN, with particular emphasis on proliferation, maturation, survival and synaptic features of abGCs as well as in hippocampal-mediated functions following acute epileptic events.

## Methods

### Animals

All animal procedures were performed in agreement with the government rules (Official Mexican Standard NOM-062-ZOO-1999) and the Animal Care and Research Advisory Committee (CICUAL, ID 10367) from Instituto de Investigaciones Biomédicas, Universidad Nacional Autónoma de México.

At the beginning of the experiment, animals aged 4–6 weeks were housed in groups of 2–4 in standard acrylic cages, maintained in a room with an inverted 12:12 light cycle, and provided with free access to water and food. Animals from both sexes were used throughout the experiments (*n* = 25 male; *n* = 26 female). *Ascl1^CreERT2^* (*Ascl1^tm1(Cre/ERT2)Jejo^/J*) mice (CreERT2) (Yang et al., 2015), and B6. Cg-*Gt(ROSA)26Sor^tm14(CAG-tdTomato)Hze^*/J (Ai14) conditional reporter line (Madisen et al., 2010) both obtained from The Jackson Laboratory were crossed to create *Ascl1^CreERT2^; CAG^FloxStopTom^ mice* and were kept in a C57Bl/6 J background. In this model, CreERT2-mediated recombination in Ascl1-expressing progenitors induces tdTomato expression in their progeny, allowing lineage tracing of Ascl1-derived cells and the analysis of their maturation and morphology in the DG.

### Genotyping of *Ascl1^CreERT2^; CAG^FloxStopTom^*

To determine the genotype of the mice, tissue samples were obtained by cutting a 1 mm segment of the tail from animals at 4 weeks of age. Tail fragments were placed in microcentrifuge tubes and processed for genomic DNA extraction using the Phire Tissue Direct PCR Master Kit (Thermo Scientific). Lysis was performed at 90 °C for 3 min, followed by a 4-min at room temperature to complete the extraction process.

Polymerase chain reaction (PCR) was performed using the Phire Tissue Direct PCR Master Kit (Thermo Scientific) with primers designed to amplify the ADN region. The primers used for the Cre recombinase detection were as follows:

- Forward: 5′-AGCCTGTTTTGCACGTTCACC-3′
- Reverse: 5′-GGTTTCCCGCAGAACCTGAA-3′

To detect the genotype of animals carrying the stop cassette that induces the expression of the TOM fluorescent protein, we used the following primers:

- Forward: 5′-CTGTTCCTGTACGGCATGG-3’
- Reverse: 5′-GGCATTAAAGCAGCGTATCC-3’

PCR amplification was performed in a thermal cycler with the following cycling conditions: initial denaturation at 98 °C for 5 min, followed by 35 cycles of 98 °C for 5 s, 60 °C for 5 s, and 72 °C for 15 s, with a final extension at 72 °C for 1 min.

PCR products were resolved on a 2% agarose gel in 1X TAE buffer, stained with GelRed (Biotium, 41003), and visualized using UV transillumination. Band sizes were compared against a DNA ladder to determine the presence or absence of each transgene.

### Induction of TOM expression in Ascl1 cells

To induce fluorescent protein Tom expression in *Ascl1^CreERT2^; CAG^FloxStopTom^* mice, we administered intraperitoneal (i.p.) injections of tamoxifen (TAM, Sigma Aldrich; >99%). Mice received a total of four injections over two consecutive days, with two injections per day spaced by 8 h. Each injection contained 120 mg/kg TAM, obtained from a 30 mg/mL stock solution dissolved in 90% corn oil and 10% absolute ethanol. All solutions were prepared 1 day prior to usage; mice were weighed prior to each TAM administration. Tamoxifen administration was completed 2 days prior to KA or saline administration in order to induce recombination in Ascl1-expressing progenitors before seizure induction.

### Intraperitoneal injection of kainic acid (KA) and induction of seizures

Kainic Acid (KA) is a compound that induces hyperexcitability primarily by acting as a potent agonist for ionotropic glutamate receptors, particularly kainate receptors, but also for AMPA/kainate receptor complexes. This leads to sustained neuronal depolarization and excessive excitatory signaling (Wang et al., 2005). KA (Sigma Aldrich; 0.75 mM) was dissolved in NaOH buffer (pH 7.4) at a concentration of 3 mg/mL. To induce two different levels of hyperexcitability, we weighed the animals prior to each injection and administered 5 mg/kg or 25 mg/kg i.p. Control animals received an average volume of saline (Sal, 0.9%) which served as the vehicle control solution. Animal behavior was recorded on video from the moment of KA administration until 3 h of post-injection. Seizure-related behavioral manifestations were classified using the Racine scale modified for mice: Racine 1 (immobility) and Racine 2 (facial automatisms), Racine 3 (forelimb movements), Racine 4 (loss of posture, spontaneous jumping, and loss of balance), and Racine 5 (generalized tonic–clonic seizures) (Racine, 1972; Sharma et al., 2018). Following this period, animals remained under observation.

### Electrode implantation and EEG recording

In an independent set of experiments, we used a group of animals to characterize the electroencephalographic (EEG) pattern and the evolution of epileptiform activity in response to the different doses of KA. EEG recordings were performed for: 5 mg/kg KA *n* = 5; (3 males; 2 females); 25 mg/k *n* = 4; (2 females; 2 males) and; Sal (*n* = 2; 1 male and 1 female). Seven days before KA administration, epidural electrodes were bilaterally implanted at the following coordinates relative to Bregma (mm): +2.0 anterior, −3.0 posterior, +1.5 right hemisphere, and −1.5 left hemisphere.

On the day of recording, electrodes were connected to The Grass Model 7 Polygraph, Grass Instrument Co, and animals were left in free movement inside an acrylic cage. EEG recording began with a 30-min habituation period, ensuring that the animal remained in a state of quiet wakefulness to minimize movement artifacts. Following habituation, a 30-min baseline recording was obtained to determine the EEG basal activity. After completing the baseline recording, we administered i.p. either dose of KA or saline (Sal) as control. EEG activity was continuously recorded for 3 h post-treatment. Simultaneously, we performed behavioral observations to record the behaviors associated with each treatment and to assign severity of seizure manifestations according to the Racine scale described in the previous section.

To assess the progression of EEG activity, follow-up recordings were performed on day 1, as well as 1, 2, and 3 weeks after the initial EEG recording, with a duration of 1 h for each time point. These follow-up recordings aimed to examine the presence of spontaneous recurrent seizures (SRS) and duration of epileptiform activity.

For EEG analysis, the three-hour recordings were segmented into 30-min intervals to measure the duration of epileptogenic events. An epileptogenic event was defined as sustained rhythmic discharges or high-amplitude spike–wave complexes. The onset of each epileptogenic event was identified by the appearance of sustained high-amplitude rhythmic activity, while the end was set when the activity showed a reduction in amplitude and frequency, returning to baseline levels or displaying a clear decrease in discharge intensity. The duration of each epileptogenic event was measured and quantified within each 30-min interval, allowing the assessment of both the intensity and temporal dynamics of epileptiform discharges throughout the recordings.

### Behavioral tests

Behavioral tests were conducted 20 days after the administration of either Sal or KA. Mice were transported to a room exclusively used for behavioral assessment. Prior to testing, a 3-day habituation and handling phase was implemented to familiarize the animals with the experimenter and minimize anxiety-related behaviors.

General locomotion was assessed using an Open Field (OF). Mice were placed in the center of a white acrylic arena (40 cm × 40 cm× 30 cm) under red light illumination at 10:00 a.m. (activity period of animals). The number of crossings within the arena was recorded over a 10-min session for two consecutive days. Behavioral activity was video recorded for subsequent analysis. Videos were analyzed offline after completion of the behavioral sessions. To prevent olfactory cues from influencing behavior, the arena was cleaned with a cleaning solution (10% EtOH and 10% Extran) diluted in distilled water between trials. The open field session also served as habituation to the arena used for the OLM task.

Following OF recording, animals were tested for the Object Location Memory (OLM) test, which requires spatial encoding and relies on the hippocampus and on the integrity of abNs (Frechou et al., 2024). A contextual cue, consisting of a black and white striped rectangle, was placed on one of the walls of an open field arena identical to the one used for open field recording. Two identical objects (Lego squared towers, 5 cm height) were positioned equidistantly from the walls (each one 10 cm from a wall) (10 cm apart one for the other) in the arena. The task consisted of two phases: the acquisition phase and the memory test phase.

During the acquisition phase, mice were placed in the center of the arena and allowed to explore both objects freely for 10 min. The time spent exploring each object was recorded and analyzed. The following day, during the memory test phase, one of the objects was moved to a novel location (10 cm from a new wall) (10 cm apart from the other object), while the other object was left in its original position. The time spent exploring both the familiar and the novel localizations was measured. Exploration was defined as the mouse making direct contact with the object using its whiskers, forepaws, or nose, but not when climbing, sitting on top of the object or exploring from the top of it. Exploration time was quantified from video recordings. Animals that explored the objects for less than 180 s during either session were excluded from the analysis. To obtain a discrimination index during acquisition, object preference was calculated as the time spent exploring either object. For statistical analysis, discrimination index (DI) calculated as: DI = ((Tnovel-Tfamiliar)/(Tnovel+Tfamiliar))*100.

During the acquisition session, both objects were identical and remained in fixed positions; therefore, DI values near zero indicated a lack of location preference. During the memory test, Tnovel corresponded to the time spent exploring the displaced object and Tfamiliar to the time spent exploring the original location. Positive DI values near or above 20 indicated preferential exploration for the novel location, whereas values near zero reflected no discrimination and negative values near or above −20 indicated preference for the familiar location (Vogel-Ciernia and Wood, 2014).

To assess a task related to neurogenesis we conducted a two-day session of single-shock Contextual Fear Conditioning (CFC) and tested memory (CFM)on the second day of training (before the shcok was delivered) and on the third day, when no shock was delivered. A single shock paradigm has been shown to be sensitive to abGC integrity (Drew et al., 2010). The task consists of pairing a shock in a given context to induce context aversion and then evaluating the aversive memory, displayed as lack of movement (except for those related to breathing) or freezing. The test was performed in a mouse conditioning chamber (25 cm × 25 cm × 20 cm) (San Diego Instruments, San Diego, CA, USA) with transparent acrylic walls and a metal rod floor. Movements were recorded using the Freeze Monitor software (Freeze Monitor, SD Instruments), which detects movement interruptions of a beam through a matrix of 16 × 16 infrared beams placed at the cage floor level. Freezing behavior was quantified automatically using Freeze Monitor software. The task was performed on day 28 after KA administration and lasted for 3 days. On the first day, each mouse was placed in the conditioning chamber and was allowed to explore freely for 170 s before receiving a single foot shock (2 s, 0.75 mA); the animal was left in the chamber for another 20s before withdrawing it from the chamber. This same process was repeated on the second day. Movement was recorded before shock administration on both trials. On day 3, mice were re-exposed to the conditioning chamber without receiving a foot shock at any time point, and movement was recorded for 180 s. The chamber was cleaned between trials. Movement was thus recorded during the first 120 s on days 1 and 2 and during 180 s on day 3. Data were analyzed as percentage of freezing time.

### Histological procedures

Thirty days after administration of KA or saline solution and 30 min after the last CFM trial, mice were deeply anesthetized with an overdose of sodium pentobarbital (210 mg/kg) and transcardially perfused with ice-cold 0.9% saline solution, followed by ice-cold 4% paraformaldehyde in 0.1 M phosphate buffer (pH 7.4). The extracted brains were post-fixed in 4% paraformaldehyde and sequentially transferred to 15 and 30% sucrose in 0.1 M phosphate buffer for cryoprotection. Coronal brain sections (40 μm thick) were obtained using a cryostat (Microm HM550, Thermo Fisher Scientific, Waltham, MA, USA) and stored in a cryoprotectant solution (25% ethylene glycol, 25% glycerol, 50% 0.2 M PB, pH 7.4) until processed for immunofluorescence.

### Immunofluorescence procedure

Immunofluorescence was performed on 40 μm free-floating coronal brain sections from the septal fraction of the hippocampus from Ascl1Cre^ERT2^; CAG^floxStopTom^ mice. Serial sections were selected at 120 μm intervals. The selected sections were washed three times in PBS (0.1 M, 0.9% NaCl, pH 7.4) for 10 min per wash, followed by permeabilization in PBS-Tx (0.3% Triton X-100) for 30 min. Sections were then immersed in a blocking solution (5% Normal Horse Serum, Vector Laboratories, Burlingame, CA, dissolved in PBS-Tx 0.3%) at room temperature for 2 h. Sections were incubated with the primary antibody anti-RFP (rabbit polyclonal, 1:500; Rockland Immunochemicals) for 48 h at 4 °C, followed by incubation with the secondary antibody Alexa Fluor 546 anti-rabbit, (1:500) (Invitrogen) or anti-rabbit Cy3 (1:250) (Jackson ImmunoResearch Laboratories) for 4 h at room temperature. The RFP antibody was used for optimal detection of Tom reporter protein. Antibodies were diluted in blocking solution. Finally, sections were stained with DAPI (1:350) for 10 min and mounted on slides using DAKO (Dako; Carpinteria, EUA) mounting medium or house made gerbatol fluorescent mounting medium to prevent bleaching.

### Confocal microscopy

#### Analysis of number of cells, cell type, and position within the DG

Images were obtained from 5 brains from the control condition (Sal), 6 brains from the 5 mg/kg dose, and 6 brains from the 25 mg/kg dose. We focused our analysis on the dorsal dentate gyrus, as this structure participates in the evaluated functions spatial processing and memory (Hunsaker and Kesner, 2008). We analyzed four serial sections per brain separated by 120 μm each, all of them contained within coordinates antero-posterior, AP: −0.94 to −2.46 mm from bregma. A confocal microscope (Nikon A1R + STORM) was used for image acquisition along the z-axis, employing a 40 × oil-immersion objective. A total of 25 to 29 optical slices were captured at 1.5 μm intervals in serial mode. Regions of interest (ROIs) where cells were more densely present across conditions were imaged. The analysis included one region from the crest, two from the suprapyramidal layer, and one from the infrapyramidal layer.

#### Substructural analysis: dendritic length and branching points, spine density, filopodia and mossy fiber boutons

We acquired images from 4 to 5 brains per condition and 5 sections per subject; using both Meta 510 and 880 LSM Airyscan confocal microscopes (Carl Zeiss, Jena, Germany).

For dendritic length and branching points measurements, images were acquired (40x; NA 1.3; oil-immersion) from 40 μm thick sections taking z stacks including 20–35 optical slices, airy unit = 1 at 0.8-um intervals. Dendritic length was then measured using the LSM Image Browser. From projections of three-dimensional reconstructions onto a single plane in granule cells expressing TOM, we traced the dendritic tree using the open free shape curve and the measure tools.

For Mossy fiber buttons (MFB), images of RFP-labeled from the CA3 region were acquired at 0.3-μm intervals (63x NA 1.4; oil-immersion) and a digital zoom of 7. Area and number of filopodia were analyzed from projections of three-dimensional reconstructions onto a single plane in the ZEN Pro 3.3 software (Zeiss, Germany). MFBs that fit the following criteria were selected for quantification: (i) the diameter of the bouton was threefold larger than the diameter of the fiber, (ii) the bouton was connected to the mossy fiber on at least one end (Toni et al., 2008). Filopodia were identified as protrusions arising from MFBs (1 mm < length < 20 mm) (Acsády et al., 1998) and number of filopodia extensions per MFB was counted.

Spines were counted manually from dendritic fragments of ~40 μm located in the middle third of the molecular layer. For image capture and analysis of morphological properties, all experimental groups under study were blind to the experimenter.

### Image analysis

#### Semi-stereological estimation of total cell numbers

The total number of cells per mm^3^ was estimated considering the imaged regions. This estimation was performed using the optical fractionator method as described by Keuker et al. (2001).:

$$N=\mathrm{\Sigma Q}*\frac{1}{\mathrm{ssf}}*\frac{1}{\mathrm{asf}}*\frac{1}{\mathrm{tsf}}$$

where *N* represents the estimated total number of cells, *Q* is the total cell count, *ssf* is the section sampling fraction, *asf* is the area sampling fraction, and *tsf* is the thickness sampling fraction.

The estimation of volume (mm^3^) for the dentate gyrus was obtained using the Cavalieri principle:

V=T∗ΣAi

where *V* is the estimated volume, *T* is the inverse of the section sampling fraction multiplied by the section thickness, and *Ai* represents the analyzed area of each section.

#### Classification of tom + adult-born granule cells (abGCs) by morphological type and position

For counting and classifying abGCs (Tom + cells), we used the Weka Segmentation 3D plugin, the 3D Counter and Analyze Particles tools in Fiji (Fiji 2.9.0 software).

To evaluate the position of the cells, we applied the method reported by Espósito et al. (2005) with some modifications. Cells were counted in the subregions of the DG including the subgranular zone (SGZ), the granular cell layer (GCL) which was divided into GCL1 (proximal to the hilus) and GCL2 (closer to the molecular layer) and the hilus (H).

For the assessment of cell phenotype, we also employed the method of Espósito et al. (2005), with some modifications. Cells were classified into different categories based on their morphology. Radial glia-like cells (RGL) were identified by the presence of a short apical process. Neural progenitor cells, categorized as Type A/B, lacked processes or displayed a horizontal orientation. Type C cells corresponded to immature neurons, characterized by a small apical process and Type D cells were classified as mature neurons, distinguished by more complex dendritic processes oriented toward the molecular layer.

### Statistical analysis

All the data are expressed as mean ± SEM. Data obtained from the quantification of Tom + cells in the different groups was assessed for normality using the Shapiro–Wilk test (*p* > 0.05). To compare the total number of Tom + cells, the proportion of cells located in different regions of the dentate gyrus (SGZ, GCL1, GCL2, and hilus), and the proportion of cells classified by phenotype and maturation phase (RGL, Type A/B, Type C, and Type D) among groups, a two-way analysis of variance (ANOVA) was performed, followed by Sidak’s *post hoc* test for multiple comparisons. Data sets analysed for ANOVA passed the normality and homogeneity criteria.

For the analysis of global EEG activity across conditions (5 mg/kg AK and 25 mg/kg AK), a Student’s t-test was conducted to compare total time of epileptiform activity (*n* = 6). For the analysis of the evolution of EEG activity, we used two-way ANOVA, followed by Tukey’s *post hoc* test for multiple comparisons (factor time, factor treatment and interaction).

For the analysis of the input (spine density) and output (mossy fiber bouton area and number of filopodia), all statistical analyses were performed with each mouse considered as the biological replicate. Because multiple measurements were obtained per mouse, such data have a nested structure. To account for this, we used a nested one-way ANOVA model with Tukey’s post hoc test, with experimental condition as a fixed effect and mouse as a random effect.

The number of crossings in the open field test and the percentage of freezing time during the memory phase of the contextual fear test were analyzed using one-way ANOVA, followed by Tukey’s post hoc test for multiple comparisons between each experimental condition. For the Object Location Memory (OLM) test analysis, one-way ANOVA, followed by Tukey’s post hoc test for multiple comparisons was applied to compare the discrimination index (DI) between locations (L1 which later became the NL and L2 which later became the FL) within each experimental group in both, acquisition and memory phases. In addition, one-sample t-tests were performed to evaluate whether DI values differed significantly from zero for each group. A *p*-value of < 0.05 was considered statistically significant. All data were analyzed using GraphPad Prism 10.1.1 software.

## Results

### Behavioral characterization of seizure severity induced by kainic acid

**Figure 1:**
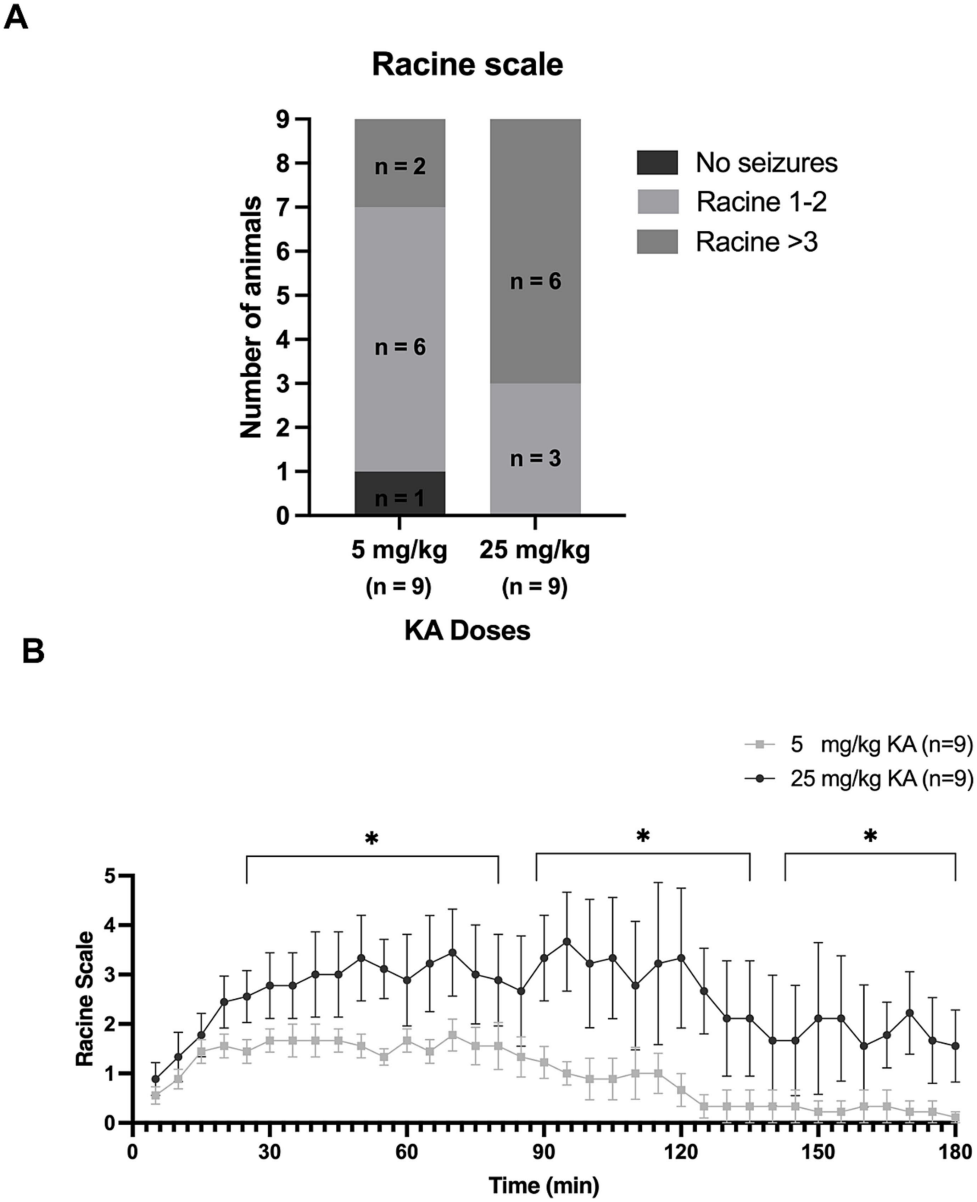
Behavioral characterization of seizure severity induced by i.p. 5 mg/kg and 25 mg/kg KA administration. **(A)** Number of animals showing no seizures, mild seizures (Racine stages 1–2), or severe seizures (Racine stage ≥3) following KA injection. **(B)** Time-course analysis of seizure severity (according to Racine scale) scored immediately after injection and for the next 3 h illustrates the behavioral progression in animals treated with 5 mg/kg KA (grey squares) (*n* = 9), or 25 mg/kg KA (black circles) (*n* = 9). * denotes *p* < 0.05 minimum statistically significant differences between 5 mg/kg and 25 mg/kg KA groups, determined by two-way ANOVA followed by Tukey’s *post hoc* test. Error bars denote SEM.

Systemic administration of kainic acid (KA) induced behavioral seizures in a dose-dependent manner. Among animals injected with 5 mg/kg KA (*n* = 9), 6/9 exhibited mild seizures corresponding to Racine 1 and 2; 2/9 animals reached Racine ≥3 and 1/9 animals did not display evident seizures. In contrast, 25 mg/kg KA administration (*n* = 9) resulted in more pronounced behavioral manifestations: 3/9 animals exhibited seizures Racine <3, whereas 6/9 animals reached Racine ≥3 (Figure 1A), indicating a stronger seizure severity. For subsequent analyses, only animals representing the predominant epileptic-like behavior within each group were included in the main figures depicting cell analysis. The rationale for including in the main analyses only those subjects that showed Racine 1–2 or Racine ≥ 3 in the low and high doses, respectively, was to reflect how the severity of seizures (and not the injected dose) would impact on cellular features and cognitive behavior. Supplementary Figure 1 presents a subcategorization of the 25 mg/kg group comparing Racine 3 and Racine 4–5 animals, since seizures were clearly more severe in Racine 4–5 and initial observations revealed evident differences in cellular distribution within the GCL between these subgroups. No experimental mortality occurred in the 5 mg/kg group, whereas 4 animals in the 25 mg/kg group died after reaching Racine 5 (data not shown). We performed a temporal analysis of seizure progression during a 3 h period after systemic KA administration. Epilepsy-related behavioral manifestations were systematically recorded and subsequently analyzed in 5 min intervals using the Racine scale. Animals injected with 5 mg/kg KA displayed Racine 1–2 manifestations which persisted for approximately 1–1.5 h before returning to baseline activity. In contrast, animals treated with 25 mg/kg KA showed behaviors corresponding to Racine 3–4, mainly during the first- and second-hour period post-injection, while Racine 1–2 persisted up to 3 h (Figure 1B). The temporal analysis of behavioral seizure progression revealed that seizure intensity increased rapidly after KA administration in the 25 mg/kg group, whereas animals injected with 5 mg/kg displayed milder and shorter events. These observations validate our model confirming a dose-dependent relationship between systemic KA administration and seizure severity and prove the use of 5 mg/kg and 25 mg/kg as representative behavioral models of mild and severe seizures, respectively.

### Electrophysiological activity reflects the behavioral severity of seizures

**Figure 2:**
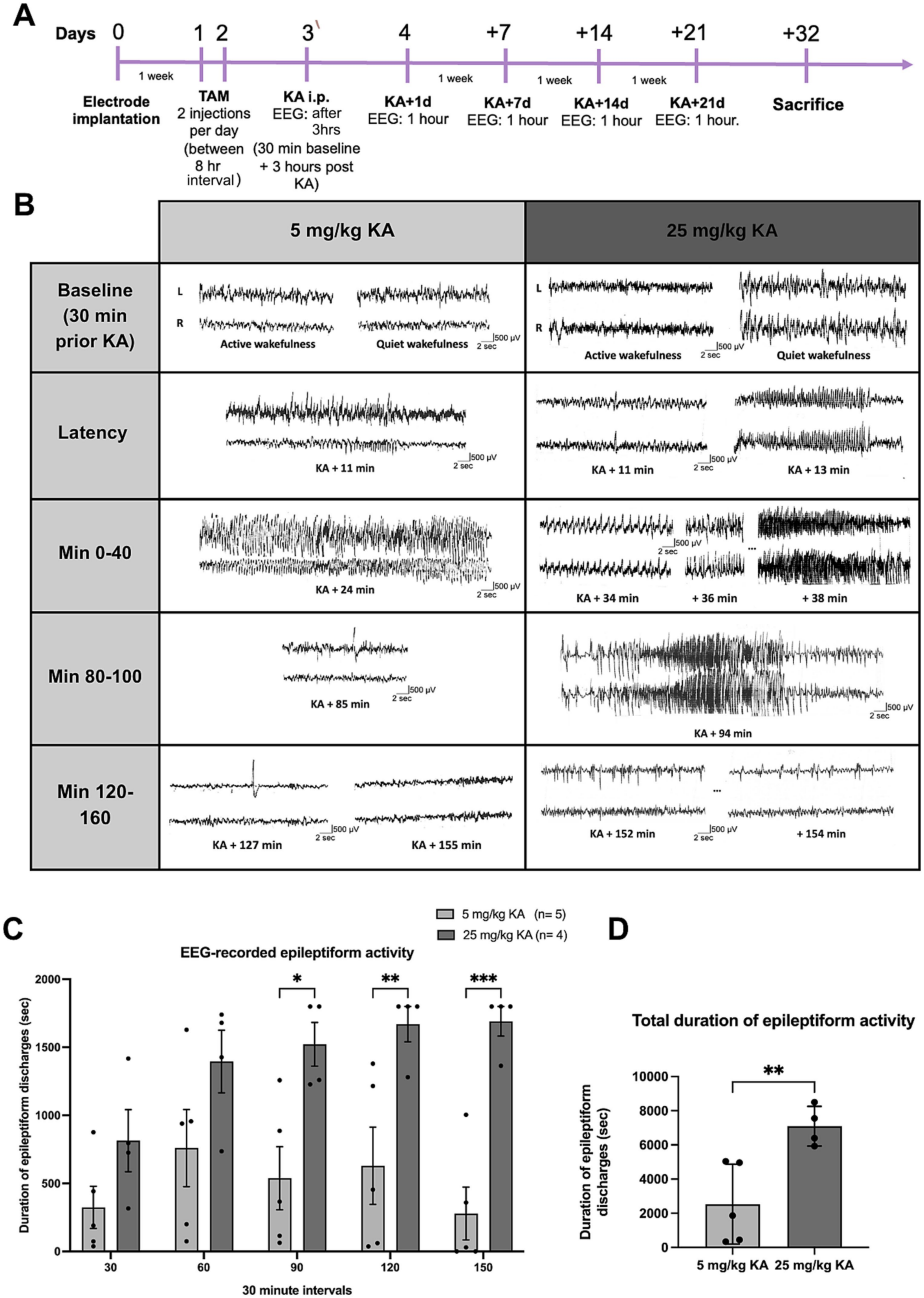
Electrophysiological characterization of seizure severity. **(A)** Timeline of EEG recordings after i.p. saline, 5 mg/kg or 25 mg/kg KA administration. **(B)** Representative cortical EEG traces from a mouse treated with 5 mg/kg KA (left panel), and a mouse treated with 25 mg/kg KA (right panel), including pre-treatment baseline, latency (referring to the time elapsed between solution administration and the onset of the first seizure) and ictal episodes. **(C)** Duration (in seconds) of epileptiform activity along 30-min intervals for 5 mg/kg and 25 mg/kg KA groups. Statistical significance of EEG activity; 5 mg/kg KA vs. 25 mg/kg KA was observed at minute 90 *p < 0.05; at minute 120 **p < 0.01 and at minute 150 ***p < 0.001 after two-way ANOVA test followed by Tukey’s *post hoc* test. **(D)** Duration (in seconds) of epileptiform activity along 3 h for 5 mg/kg and 25 mg/kg KA groups. Statistical significance of total duration of epileptiform activity; 5 mg/kg KA vs. 25 mg/kg KA was observed **p < 0.01 after t unpaired test. Data are expressed as mean ± SEM. Scale: [500 μV, 2 s].

To identify the electroencephalographic (EEG) patterns associated with seizure severity, we performed EEG recordings in a different set of animals on the same day when i.p. saline or KA was administered and at 1, 7, 14, and 21 days post-injection (Figure 2A). Supplementary Figure 2 provides additional detail on EEG traces and duration of epileptiform activity. The saline group showed regular cortical activity without epileptiform discharges throughout the recording period (Supplementary Figure 2A). In contrast, animals administered with 5 mg/kg of KA displayed short, intermittent epileptiform events, whereas those receiving 25 mg/kg KA exhibited continuous, high-amplitude ictal discharges of prolonged duration (Figure 2B; for more representative traces see Supplementary Figure 2B). A clear association was observed between the intensity of EEG activity and the severity of epileptic-associated behavior previously described. The latency to the first seizure averaged 11 min post-injection in the 5 mg/kg KA group and 6 min in the 25 mg/kg KA group, indicating an earlier presentation of epileptiform activity at the higher dose. In the 5 mg/kg KA group, EEG activity gradually declined toward baseline levels within 3 h, whereas in the 25 mg/kg KA group, epileptiform discharges persisted throughout the entire recording period. Importantly, epileptiform activity was restricted to the day of administration, as no spontaneous recurrent seizures (SRS) were detected during subsequent weekly recordings (data not shown). Quantitative analysis of epileptiform activity duration was performed at 30 min intervals across a 150 min recording period (Figure 2C). The statistical analysis considered “time” (30, 60, 90, 120, and 150 min), “treatment” (5 mg/kg vs. 25 mg/kg KA), and their interaction. A two-way ANOVA revealed a significant main effect of treatment (****p < 0.0001), whereas the effect of time and treatment × time interaction were not statistically significant. During the first 30 and 60 min, differences in the duration of discharges between groups were not statistically significant, although the 25 mg/kg KA group tended to display evident longer activity (30 min: 814 ± 227.5 s; 60 min: 1396 ± 230.1 s) compared with the 5 mg/kg KA group (30 min: 324 ± 155.1 s; 60 min: 759.8 ± 283.5 s). At 90 min, a significant difference was observed between groups with the 25 mg/kg group showing longer discharges (1,522 ± 160.7 s) than the 5 mg/kg group (538 ± 231.5 s; two-way ANOVA, treatment effect, *p < 0.05). This difference persisted through the remaining intervals (5 mg/kg KA: 120 min = 629.6 ± 283.9 s; 150 min = 278.8 ± 193.6 s; 25 mg/kg KA: 120 min = 1,670 ± 130 s; 150 min = 1,691 ± 109 s), with the 25 mg/kg KA group continuing to display significantly longer discharges than the 5 mg/kg KA group at 120 min (**p < 0.01) and 150 min (***p < 0.001) (Supplementary Figure 2C, Table 1). The total duration of epileptiform activity during the 2.5 h session was significantly greater in the 25 mg/kg KA group than in the 5 mg/kg KA group **p < 0.001 (Figure 2D).

Together, these findings demonstrate that electrophysiological activity is related to the administered KA dose. Animals receiving 25 mg/kg KA exhibited earlier onset, longer duration, and sustained epileptiform discharges compared to those treated with 5 mg/kg KA, confirming a dose-dependent relation between KA concentration and EEG activity.

### Systemic kainic acid administration does not alter the total number of Asc-1-derived cells in the dentate gyrus

**Figure 3:**
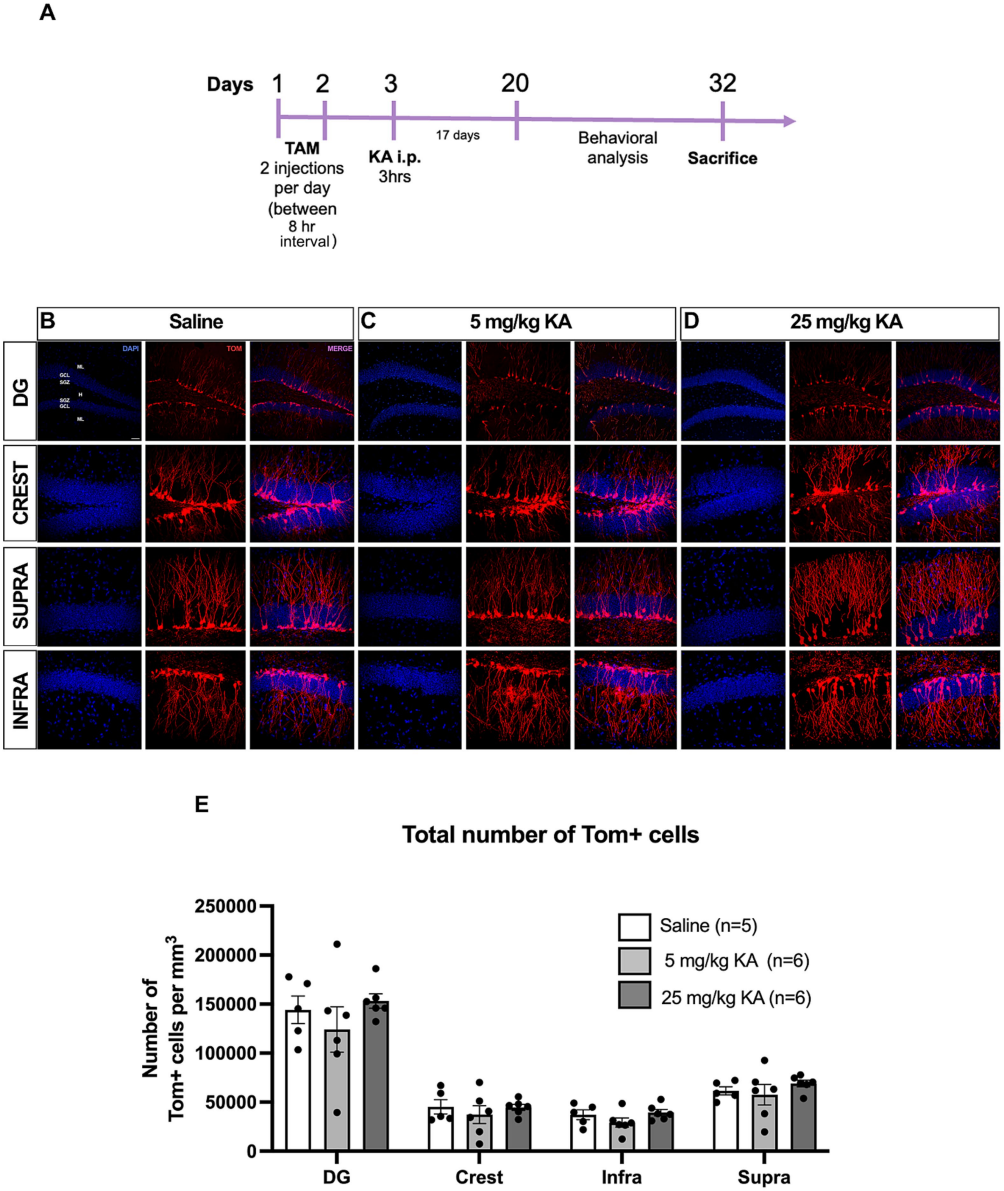
Semi esterological estimation of abGCs in the dentate gyrus following saline or systemic KA. **(A)** Experimental timeline. **(B–D)** Representative confocal images of Tom + cells in the DG from Sal **(B)**, 5 mg/kg KA **(C)**, and 25 mg/kg KA administered **(D)** animals. **(E)** Estimated total number of Tom + cells per mm^3^ within the whole analysed volume of dorsal DG as well as in the crest, suprapyramidal, and infrapyramidal regions. Cell counts were obtained via semi-stereological methods. Non statistical significant differences between conditions were observed after two-way ANOVA test followed by Sidak’s *post hoc* test. Error bars denote SEM. *n* = 5 (Sal), *n* = 6 (5 mg/kg KA), *n* = 6 (25 mg/kg KA). Scale bar: 50 μm.

To evaluate whether systemic KA administration-induced hyperexcitability altered the generation of adult-born granule cells (abGCs) in the dentate gyrus (DG), confocal microscopy analyses were performed in the dorsal hippocampi of *Ascl1^CreERT2^; CAG^FloxStopTom^* animals at 30 d after administration of saline, 5 mg/kg KA, or 25 mg/kg KA (Figure 3A). High-resolution panoramic images of the dorsal DG (−1.3 to −2.8 mm from Bregma) were obtained using confocal microscopy (20X) to assess the overall Ascl1 population and its progeny, and then specific regions of interest were analyzed in the crest, suprapyramidal, and infrapyramidal blades at 40X for each condition (Figures 3B–D).

Semi-stereological quantification of tdTomato (Tom^+^) cells within the analyzed volume of dorsal DG (see methods) revealed no statistically significant differences among saline, 5 mg/kg KA, and 25 mg/kg KA treated animals (Figure 3E; two-way ANOVA revealed no effect of treatment, subregion, or treatment × subregion interaction; all *p* > 0.05). Separate analyses of the crest, suprapyramidal, and infrapyramidal subregions also showed statistically similar cell numbers among conditions.

Together, these findings indicate that acute seizure activity, regardless of intensity, did not significantly modify the total number of abGCs derived from Ascl1 progenitors in the DG.

### Seizure severity modulates the distribution and maturation of abGCs in the dorsal dentate gyrus

**Figure 4:**
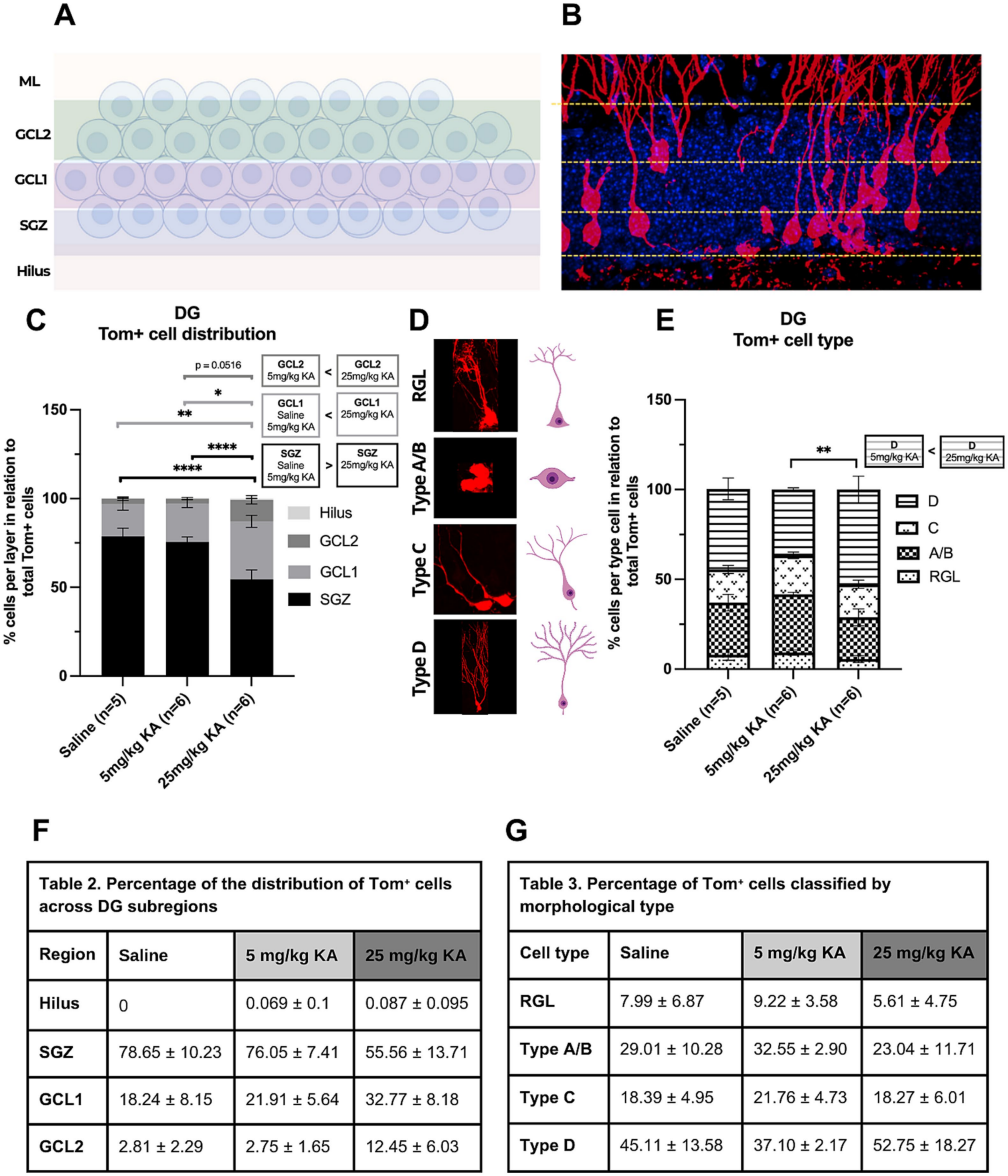
Seizure severity modulates the distribution and maturation-related features of Tom+ cells in the DG. **(A)** Schematic diagram of DG subdivisions: subgranular zone (SGZ), inner granular layer (GCL1), outer granular layer (GCL2), and hilus. **(B)** Representative image showing the subregions of the DG used for estimations. **(C)** Proportional analysis of Tom + cell distribution within DG subregions in saline, 5 mg/kg KA, and 25 mg/kg KA treated mice. Statistical significance for KA conditions and of abGCs per layer: in SGZ for Sal vs. 5 mg/kg KA and 5 mg/kg KA vs. 25 mg/kg KA ****p < 0.0001 and in GCL1 for Sal vs. 25 mg/kg KA **p < 0.01 and 5 mg/kg KA vs. 25 mg/kg KA *p < 0.05 after two-way ANOVA test followed by Sidak’s *post hoc* test. Error bars denote SEM. **(D)** Morphological classification of Tom + cells: RGL (radial glia-like), Type A/B (progenitors), Type C (immature), and Type D (mature granule neurons). **(E)** Proportional analysis of each cell type in saline, 5 mg/kg KA, and 25 mg/kg KA groups. Statistical significance for KA conditions and % of type cell: for D type cell for 5 mg/kg KA vs. 25 mg/kg KA **p < 0.01 after two-way ANOVA test followed by Sidak’s *post hoc* test. Error bars denote SEM. **(F)** Percentage distribution of Tom^+^ cells in DG subregions following saline or KA administration. **(G)** Percentage of Tom^+^cells classified by morphological type in the DG following saline or KA administration. Data are expressed as mean ± SEM. n = 5 (Sal), n = 6 (5 mg/kg KA), n = 6 (25 mg/kg KA).

The correct positioning of abGCs within the granule cell layer (GCL) is essential for establishing appropriate connections with pre-existing hippocampal neurons and serves as an indicator of neuronal maturation (Espósito et al., 2005). To evaluate whether systemic acute KA administration-induced epileptiform activity affected the positioning and maturation of abGCs, we analyzed the spatial distribution and phenotype of Tom^+^ cells in the DG of animals administered with saline, 5 mg/kg, or 25 mg/kg of systemic KA. A schematic representation was generated to illustrate the regions of the DG analyzed for quantification: SGZ, GCL1 and GCL2, and hilus along with a representative confocal image showing these corresponding subdivisions (Figures 4A,B).

Figure 4C shows that in saline and 5 mg/kg KA-treated animals, the majority of Tom^+^ cells were located within the SGZ (78.65% ± 10.23 and 76.05% ± 7.41, respectively), while in animals treated with 25 mg/kg KA, the proportion of Tom^+^ cells in the SGZ was significantly reduced (55.56% ± 13.71; two-way ANOVA revealed a significant treatment × layer interaction, ****p < 0.0001; Šidák’s *post hoc* test: saline vs. 25 mg/kg KA, ****p < 0.0001; 5 mg/kg KA vs. 25 mg/kg KA, ****p < 0.0001). In line with this, the proportion of Tom^+^ cells located in GCL1 was significantly higher in the 25 mg/kg KA group (32.77% ± 8.18) compared to both saline (18.24% ± 8.15, **p < 0.01) and 5 mg/kg KA groups (21.91% ± 5.64, *p < 0.05). Although a trend toward a higher percentage of Tom^+^ cells was observed in GCL2 in the 25 mg/kg KA group (12.45% ± 6.03), the difference was not significant; however, the proportion was higher compared to saline (2.81% ± 2.29) and 5 mg/kg KA (2.75% ± 1.65, *p* = 0.0516) (Figure 4F, Table 2). No significant differences were detected in the distribution of Tom^+^ cells within the different layers of the DG between saline and 5 mg/kg KA groups (Figures 4C,F, Table 2). These results indicate that the positioning of abGCs, migration from the SGZ toward the granular layers, was affected only in animals administered with 25 mg/kg but not in those receiving 5 mg/kg. The severity of seizure display (Racine 3 or 4/5) in animals receiving 25 mg/kg affected the migration profile for the SGZ in correspondence to seizure display (Supplementary Figure 1).

Because the morphology of abGCs is also closely related to their maturation state (Espósito et al., 2005), we next classified Tom^+^ cells according to four distinct morphologies corresponding to their type and progressive neurogenic stage: (1) RGL, radial glia-like cells characterized by a long apical process with short branches; (2) Type A/B, progenitor-like cells with elongated somata and short lateral processes parallel to the GCL; (3) Type C, immature neurons with a small apical dendritic tree extending toward the molecular layer; and (4) Type D, mature granule neurons with a compact soma with a radially oriented large dendritic tree extending into the molecular layer (Figure 4D). Two-way ANOVA revealed a significant treatment × cell type interaction (*p = 0.0371) as well as a significant effect for cell type (****p < 0.0001). *Post hoc* comparisons show that animals treated with 25 mg/kg KA exhibited a higher proportion of mature type D cells (52.8% ± 18.3) compared to the 5 mg/kg KA group (37.1% ± 2.2; Šidák’s test, *p < 0.05), whereas no differences were detected relative to saline-treated animals (45.1% ± 13.6) (Figures 4E,G). These results suggest that intense seizure activity may promote neuronal maturation features. Interestingly, seizure severity, independent of KA concentration, impacted abGC characteristics. Among animals receiving 25 mg/kg KA, a natural subdivision was observed: three animals displayed Racine 3 epileptic-like behavior, while another three displayed Racine 4–5 behavior (see Supplementary Figure 1). Comparisons within the 25 mg/kg group revealed that changes in cell distribution within the GCL and in maturation type were more pronounced in animals with severe seizures than in those with milder seizures.

Collectively, these findings indicate that while the total number of abGCs in the DG remains unaltered after acute seizures associated with systemic KA, seizure severity significantly influences their spatial distribution within the granule cell layer and has an impact on maturation, at least as suggested by their morphology.

### Systemic kainic acid concentration differentially modulates connectivity features of abGCs at the input and output levels

**Figure 5:**
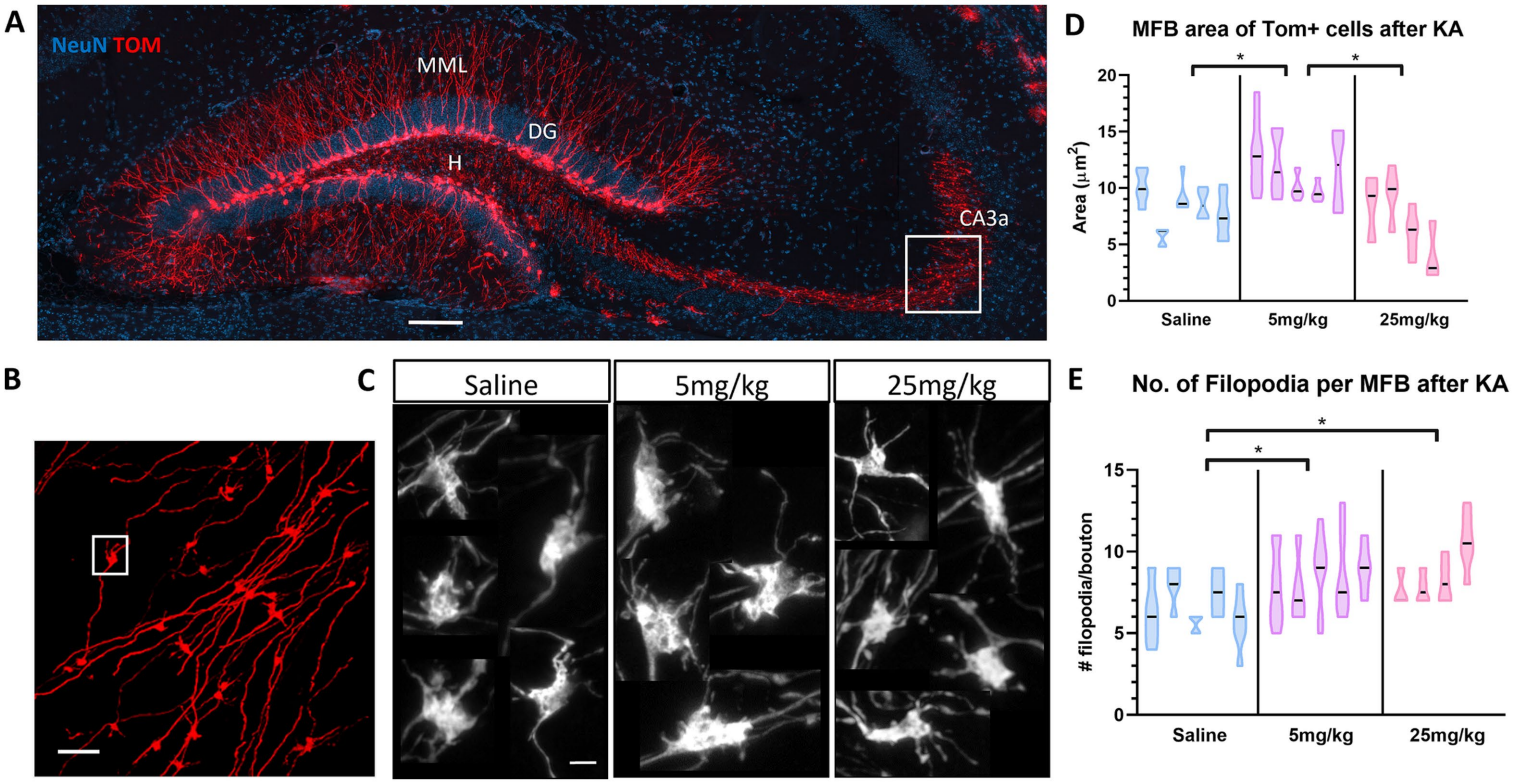
Effects of systemic KA injections at the output level of Tom+ abGCs. **(A)** Panoramic view of the DG and CA3 for a Sal animal, from where morphological analyses were performed; NeuN (blue), Tom (red); inset CA3a. H: hilus, MML: medial molecular layer, DG: dentate gyrus. Scale bar 150 μm. **(B)** Representative image of CA3a mossy fiber boutons (MFB) from Tom + +abGcs. Inset: example of MBF. Scale bar 40 μm. **(C)** Representative confocal images of MFBs in saline and KA-treated groups. 4–5 Individual boutons cropped from the original images are shown. Scale bar 5 μm. **(D)** MFB area was measured in 15 to 35 terminals from different axons (4 to 5 mice per condition). Statistical significance for Sal vs. 5 mg/kg and for 5 mg/kg vs. 25 mg/kg *p < 0.05. Error bars denote SEM. **(E)** Number of filopodia in MFBs was measured in the same 15 to 35 terminals evaluated for the analysis of MFB area. Statistical significance for Sal vs. 5 mg/kg and for Sal vs. 25 mg/kg *p < 0.05 after nested one-way ANOVA followed by Tukey’s *post hoc*. Data are expressed as mean ± SEM.

In physiological conditions, granule cells establish contacts with pyramidal cells in CA3 through their terminals, the mossy fiber boutons (MFB), and recruit GABAergic feedforward inhibition on pyramidal cells via filopodia extended from granule neuron terminals (Acsády et al., 1998; Restivo et al., 2015; Toni et al., 2008). To get further insights into the circuit connectivity and organization analysis of Tom + cells born in the DG after hyperexcitability was induced, we explored some morphological features of their input and output in animals that received either saline, 5 mg/kg or 25 mg/kg KA. We obtained confocal images from MFBs in CA3a (Figure 5A) and measured their area, as well as the number of filopodia per terminal as indicators of structural plasticity at a postsynaptic level. To account for the nested structure of the data, all analyses were performed using nested one-way ANOVA with mouse as a random effect. MFB area showed a significant effect of treatment (*p* < 0.05) in Tom + cells. Variance component analysis revealed that variability within each mouse-MFBs (SD = 2.156; variance = 4.647) exceeded variability between mice (SD = 1.510; variance = 2.280). This corresponded to an intraclass correlation coefficient of 0.33, indicating that 33% of the total variance is attributable to differences between mice. *Post hoc* comparisons indicated that MFB area increased from approximately 7μm^2^ in animals in the Sal group to 11μm^2^ in the 5 mg/kg group (*p* < 0.05, one-way ANOVA followed by Tukey’s *post hoc*) (Figures 5C,D). In contrast, the 25 mg/kg KA group exhibited MFB areas comparable to Sal (7um^2^) but significantly smaller than those in the 5 mg/kg KA group (*p < 0.05, one-way ANOVA test followed by Tukey’s *post hoc*). (Figures 5C,D). Regarding the number of filopodia, we also detected a significant effect of treatment (*p* < 0.05). In this case, variability within each mouse filopodia (SD = 1.818; variance = 3.305) was greater than between mice variability (SD = 0.704; variance = 0.495), yielding an intraclass correlation coefficient of 0.13. This indicates that 13% of the variance is explained by differences between mice, consistent with the non-significant nested term and suggesting a relatively homogeneous structure across animals. The Sal group displayed an average of 4 filopodial extensions, suggesting a mature phenotype. This number increased significantly to approximately 9 extensions per MFB after systemic administration of either 5 mg/kg or 25 mg/kg groups (*p < 0.05 for 5 mg/kg and 25 mg/kg after one-way ANOVA followed by Tukey’s *post hoc*) (Figure 5E) suggesting that cells developing in a hyperexcitable milieu increase the number of structures implicated in synaptic inhibition at the output level.

**Figure 6:**
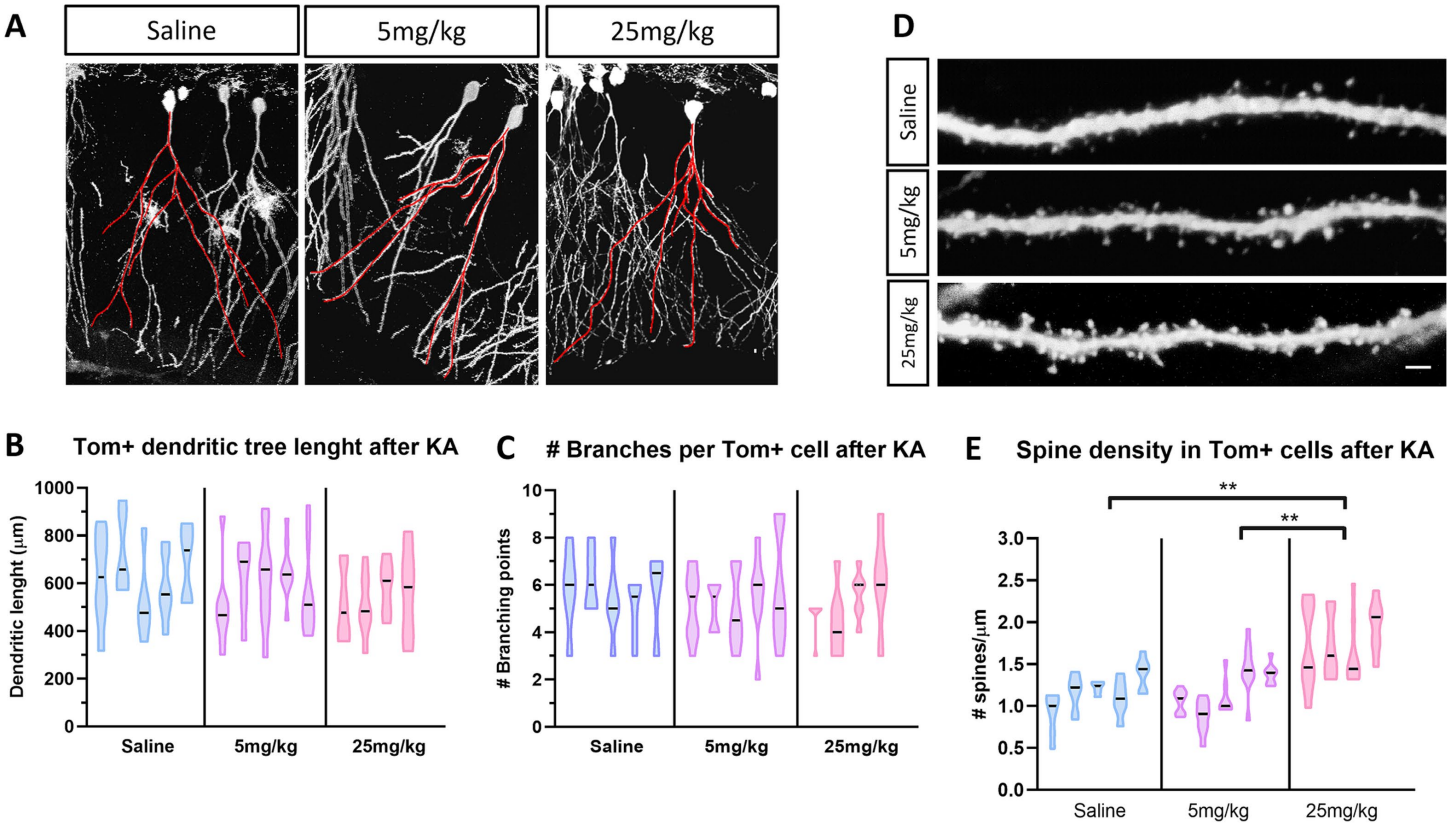
Effects of systemic KA injections on dendrites from Tom+ abGCs. **(A)** Representative images from Tom + abGCs after saline and KA injections. Red lines trace the dendritic arbor of individual Tom + neurons. Scale bar 20 μm. **(B)** Quantification of dendritic complexity measured by dendritic length and **(C)** branching points in KA and Sal conditions. Data were obtained from 25 to 42 neurons (4 to 5 mice per condition). **(D)** Dendritic fragments from Tom + abGCs in saline and KA injections. Scale bar 2 μm. **(E)** Spine density measured from 29 to 45 neurons (4 to 5 mice per condition) in saline and KA injections. Statistical significance for Sal vs. 25 mg/kg and for 5 mg/kg vs. 25 mg/kg ** *p* < 0.01 after nested one-way ANOVA followed by Tukey’s *post hoc*. Data are expressed as mean ± SEM.

Imaging and tracing of the dendritic tree of Tom + abGCs showed that the length of the dendritic tree was similar among groups (Figures 6A,B). We also measured the number of branching points in the dendritic tree as a measure of complexity, but no change was observed either (Figure 6C). Finally, as a measure of input connectivity we quantified the dendritic spine density in the middle fraction of the dendrites of the Tom + from all groups. We observed a significant effect of treatment (***p < 0.0001) in the dendritic spine density in the middle fraction of the dendrites of the Tom + from all groups. Variance component analysis showed that variability within each mouse spine density (SD = 0.273; variance = 0.0746) exceeded between mice variability (SD = 0.171; variance = 0.0293), corresponding to an intraclass correlation coefficient of 0.28. Thus, 28% of the total variance is attributable to differences between mice, supporting the inclusion of the nested structure. Post hoc analysis revealed a significant increase in spine density exclusively in the 25 mg/kg group (1.75 spines/μm) compared to Sal (1.15 spines/μm) and 5 mg/kg (1.17 spines/μm) groups (**p < 0.001, one-way ANOVA followed by Tukey’s *post hoc*) (Figures 6D, E) suggesting an increase in the input connectivity at higher doses of systemic KA administration. Together, these results suggest a differential response at the input and output levels from neurons born in different hyperexcitable contexts.

### Systemic kainic acid administration impairs long term place recognition, but not contextual fear memory

Several studies have pointed at alterations in spatial memory associated to intrahippocampal kainic acid injections in mice and rat, which provoke temporal lobe epilepsy or hippocampal damage (Hernández-Mercado and Zepeda, 2022; Van Den Herrewegen et al., 2019). To assess if hyperexcitability induced by KA equally affected behavior, we analyzed the performance of animals administered with Sal, 5 mg/kg, and 25 mg/kg KA.

Our first approach was to evaluate locomotion in the open field, as the two cognitive tasks that followed required the animals to move regularly.

**Figure 7:**
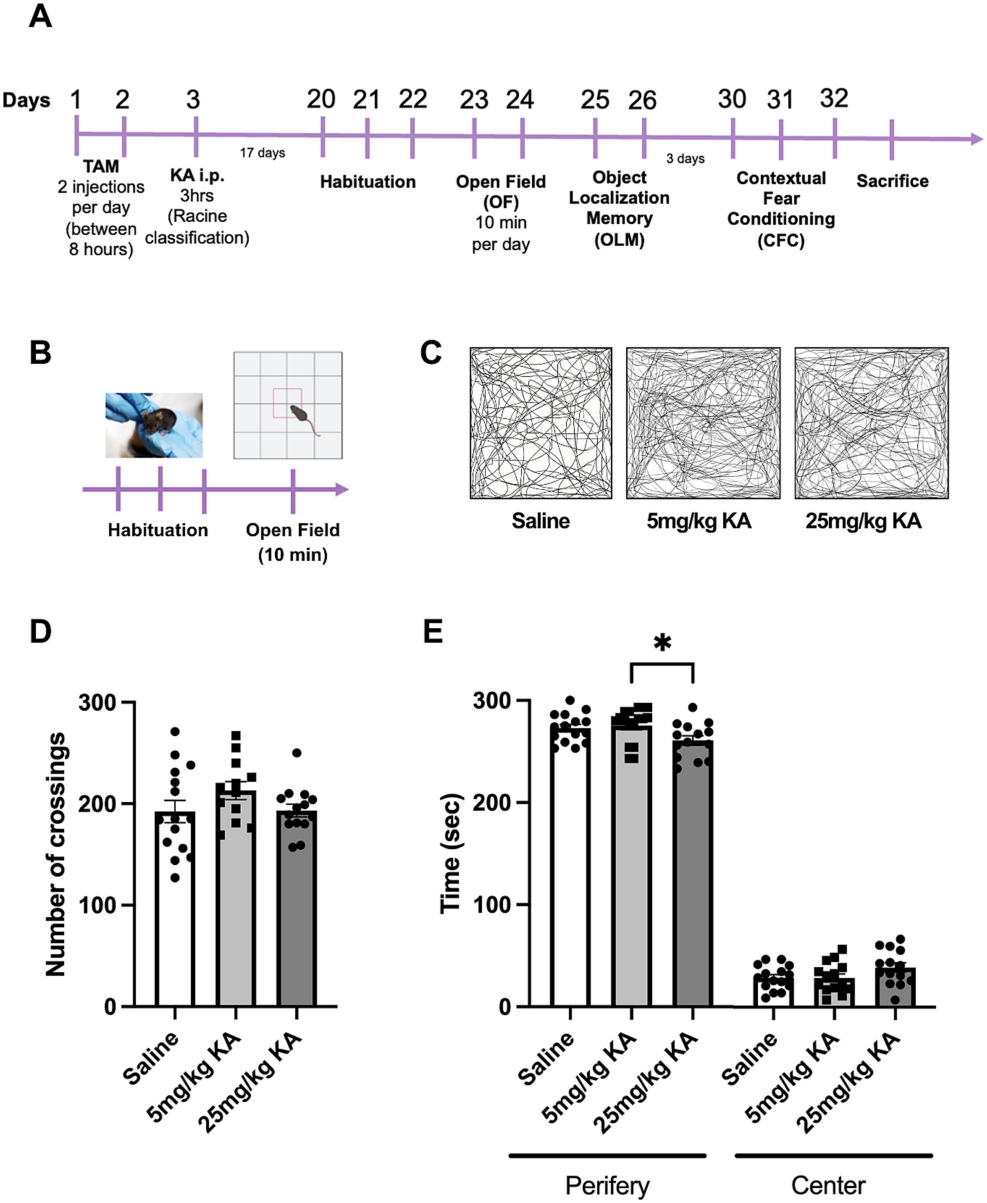
Locomotor activity following seizure induction. **(A)** Experimental timeline. **(B)** Schematic of the open field test (OF) protocol. **(C)** Representative locomotor trajectories during the 10 min session across experimental conditions. **(D)** Total number of quadrant crossings. **(E)** Time spent in central versus peripheral quadrants: 5 mg/kg KA vs. 25 mg/kg KA *p < 0.05 after one-way ANOVA followed by Tukey’s post hoc test. Data are expressed as mean ± SEM. *n* = 15 (Sal), *n* = 12 (5 mg/kg KA), *n* = 13 (25 mg/kg KA).

Figure 7A shows the experimental timeline focusing on behavioral analysis. Figure 7B shows representative traces from animals administered with Sal (left), 5 mg/kg (center), and 25 mg/kg (right). The number of total crossings in the arena was similar among groups as shown by one-way ANOVA (Figure 7C), while animals from the 25 mg/kg group showed a significant decrease in peripheral crossings (260.9 ± 17.3; one-way ANOVA, *p < 0.05; Tukey’s *post hoc* test) compared to Sal (272.9 ± 14.17), and 5 mg/kg (275.3 ± 16.85) and without affecting central ones. These results provided confidence that any of the groups presented hyperlocomotion or hypolocomotion.

**Figure 8:**
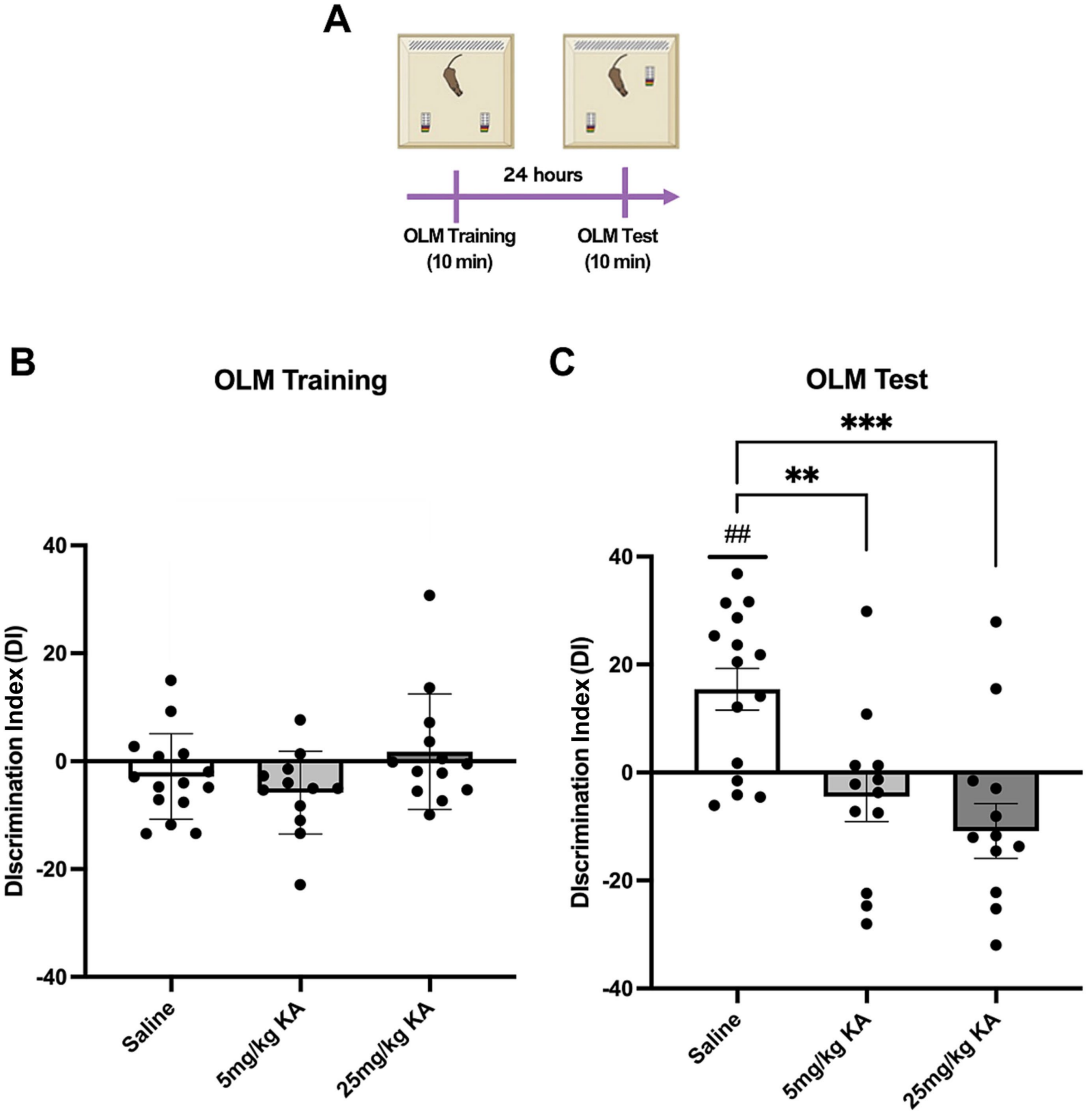
KA disrupts spatial discrimination performance in the OLM task. **(A)** Schematic diagram of the OLM paradigm illustrating the training and test session separated by a 24 h interval. **(B)** Discrimination index (DI) during OLM training. All groups displayed DI values near zero, indicating absence of baseline location preference. Error bars denote SEM. **(C)** DI during OLM test. Saline-treated mice showed robust preference for the novel location, whereas mice receiving 5 mg/kg or 25 mg/kg KA exhibited significantly different DI values, reflecting impaired spatial localization memory. Statistical significance for KA conditions in the test session: Saline vs. 5 mg/kg KA **p < 0.01 and Saline vs. 25 mg/kg KA ***p < 0.001 after one-way ANOVA followed by Tukey’s *post hoc* test. The horizontal line above the saline group (##) indicates that DI values were significantly different from chance level (DI = 0; one-sample *t* test, *p* < 0.01). Data are expressed as mean ± SEM. n = 15 (Sal), *n* = 12 (5 mg/kg KA), *n* = 13 (25 mg/kg KA).

Next, we analyzed if our KA schemes affected object location and contextual fear memory. We first analyzed the performance of animals in the object location test (Figure 8A) and observed that all animals displayed a similar discrimination index (near to 0) for the objects in the training phase (Figure 8B; Saline: - 2.847 ± 7.93, 5 mg/kg KA: - 5.376 ± 7.67, 25 mg/kg KA: 1.74 ± 10.71). However, in the test day, where one of the objects was displaced to a new location, only Sal animals showed preference for this location (15.43 ± 14.92; one-sample *t* test: t(14) = 4.006, **p < 0.01), whereas animals that received 5 mg/kg (−4.47 ± 15.95; one-sample t test: t(11) = 0.972, *p* = 0.3551) and 25 mg/kg (−10.83 ± 18.32; one-sample *t* test: t(12) = 2.13, *p* = 0.053) KA did not show DI values significantly different from zero, indicating no preference for the novel location. In addition, both KA-treated groups showed a significantly lower preference for the novel location compared with the saline group (Figure 8C; one-way ANOVA, *F*(2,37) = 9.873, *p* = 0.0004; Tukey’s *post hoc* test: Saline vs. 5 mg/kg KA, *p < 0.05; Saline vs. 25 mg/kg KA, **p < 0.01), indicating a deficit in novelty recognition.

**Figure 9:**
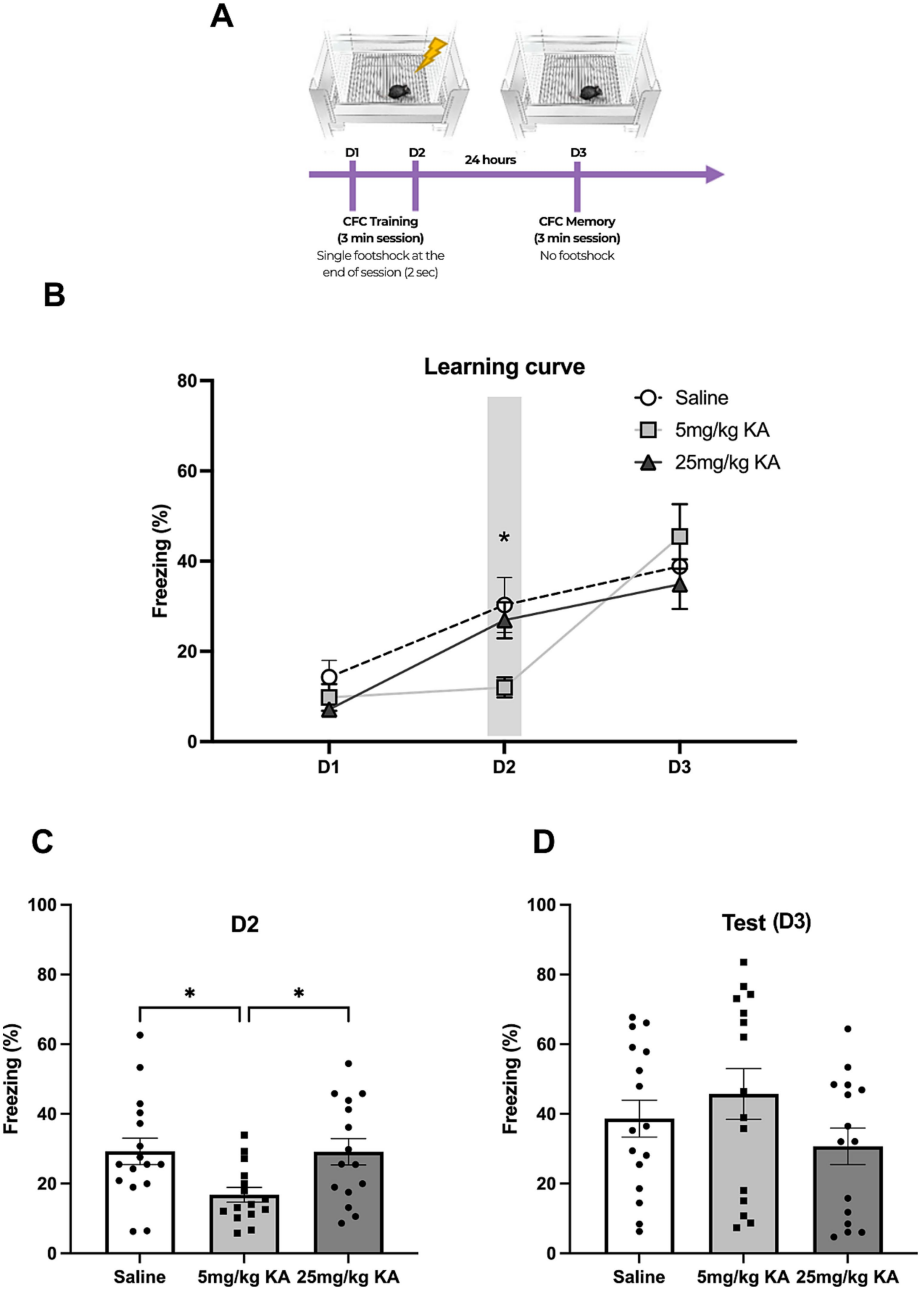
Contextual fear conditioning performance. **(A)** Schematic diagram of the CFC paradigm showing the single-shock training session and the memory test 24 h later. **(B)** Learning curve across training days (D1–D3). Saline- and 25 mg/kg KA treated mice showed a progressive increase in freezing behavior, whereas 5 mg/kg KA-administered mice showed a learning delay as they displayed a similar % in freezing time as on Day 1 (* *p* < 0.05 vs. Sal and 25 mg/kg KA at D2; gray box indicates this time point). **(C)** Freezing behavior on D2. Animals from the 5 mg/kg KA group exhibited significantly lower freezing compared to the saline and 25 mg/kg groups (*p < 0.05). **(D)** Freezing levels during the memory test. No significant differences were observed among groups. Data are expressed as mean ± SEM. *n* = 16 (Sal), *n* = 14 (5 mg/kg KA), *n* = 15 (25 mg/kg KA).

To further evaluate another task that has been shown to relate to neurogenesis (Besnard and Sahay, 2021; Drew et al., 2010), we analyzed the performance of animals in contextual fear conditioning and memory (Figure 9A). Our results show that animals that received 5 mg/kg KA exhibited a delayed acquisition of the task, as they froze significantly less than Sal on the second day of the test, when memory was already evaluated (Figures 9B, C; Sal: 29.71 ± 3.58, 5 mg/kg KA: 16.82 ± 2.13, 25 mg/kg KA: 29.16 ± 3.76; one-way ANOVA, *p* = 0.0126; Tukey’s post hoc: Sal vs. 5 mg/kg KA, *p < 0.05; 5 mg/kg KA vs. 25 mg/kg KA, *p < 0.05). However, these animals reached control levels of freezing by the third day of testing, after receiving a reinforcement of the aversive stimulus (administered at the end of days 1 and 2) (Figure 9D; Sal: 36.68 ± 5.27, 5 mg/kg KA: 44.07 ± 7.59, 25 mg/kg KA: 34.90 ± 5.49; one-way ANOVA, *p* = 0.582). Surprisingly, animals that received 25 mg/kg showed a learning curve similar to Sal treated animals (Figures 9B,C) and also showed similar levels of freezing in the test session (day 3) (Figures 9C,D) thus suggesting that no affection in this type of memory took place or that a compensatory effect may have occurred.

## Discussion

In the present study, we investigated how two levels of acute hyperexcitability shape adult hippocampal neurogenesis (AHN) and how this is associated with hippocampal-dependent memory performance. We used the *Ascl1^CreERT2^; CAG^FloxStopTom^* mice lineage-tracing strategy and performed a systemic administration of i.p. KA that induces mild or severe epileptic seizures in the absence of recurrent spontaneous seizures. Thus, we were able to examine the effects of seizure severity independently from those previously associated with chronic epileptogenesis. Our results demonstrate that, at 30 days post-injection (dpi), acute seizures, regardless of severity, do not affect the overall structure of the dentate gyrus nor the number of adult-born granule neurons (abGCs) observed in this same structure. However, seizure severity selectively influenced the spatial positioning and some maturation features of abGCs while inducing alterations in the synaptic structures of new neurons. Alterations on synaptic structures were not generalized. Our findings rather reveal a dissociation between global dendritic architecture, postsynaptic spine organization, and presynaptic output remodeling, indicating that acute seizure-induced hyperexcitability induces compartment-specific and dose-dependent forms of structural plasticity rather than uniform neuronal growth or degeneration.

On a behavioral level, spatial memory showed to be particularly susceptible to become altered as a result of hyperexcitability as both types of acute seizures induced a deficit in location recognition, but not in contextual fear learning and memory.

Together, our findings suggest that the varied consequences of seizure severity on adult hippocampal neurogenesis are more qualitative than quantitative, and differentially affect morphological traits, positioning, and potentially circuit connectivity, which would in turn affect selective hippocampal-related functions.

### Seizure severity and the long-term regulation of adult neurogenesis

The absence of a significant increase in abGC number at 30 dpi following either 5 mg/kg or 25 mg/kg KA injection is consistent with previous work reporting that seizure-induced increases in neurogenesis occur after a prolonged period of seizure activity (Mohapel et al., 2004; Parent et al., 1997).

Our results show that KA concentrations used in this work were not sufficient to induce a significant increase in the number of abGCs as they may not have survived long enough or seizures may not have had the frequency or intensity observed in status epilepticus to drive the increase reported in other studies (Mohapel et al., 2004; Parent et al., 1997). Therefore, milder hyperexcitability states as those produced in our work do not lead to a sustained aberrant increase of abGCs.

Importantly, we could neither detect a reduction in the number of abGCs, suggesting that the levels of hyperexcitability induced here were not sufficient to compromise the activity of the progenitor pool, nor to induce a decrease in abGCs survival, even when they develop in a hyperexcitable context. In chronic epilepsy models, KA-induced severe and recurrent seizures drive radial glia-like cells (RGLs) towards terminal differentiation and reactive astrocytosis due to their repeated activation, resulting in a depletion of progenitor cells in the long-term (Sierra et al., 2015). Although we did not evaluate astrogliosis, our observations suggest that the neurogenic capacity of progenitor cells remained preserved as well as the ability of the abGCs to survive in a hyperexcitable context, given that the overall number of abGCs did not change, which further underscores the importance of seizure duration and recurrence in dictating the long-term neurogenic potential of progenitor cells.

### Severe acute seizures disrupt laminar position and are associated with a higher proportion of abGCs displaying mature morphological features

Although total neurogenesis was preserved, severe acute seizures (Racine >3) induced pronounced changes in the laminar positioning of abGCs within the granule cell layer (GCL), as a higher number was found in the middle of the GCL or even in the most proximal portion to the internal molecular layer. Proper positioning of abGCs is essential for their integration into hippocampal circuitry. Under physiological conditions, abGCs migrate from the subgranular zone (SGZ) toward the inner and middle portions of the GCL, reaching their final stable position by approximately 4 weeks after birth, coincident with the acquisition of functional glutamatergic synapses and complete maturation (Espósito et al., 2005).

Consistent with our findings, previous models of epilepsy have reported abnormal dispersion and distribution of immature-DCX + and mature abGCs across the GCL (Jessberger et al., 2005; Moura et al., 2021). Such results were obtained from chronic or severe hyperexcitability events. Our results show that even milder seizures such as those provoked by 25 mg/kg, but not by 5 mg/kg may result in abnormal positioning of abGCs underscoring the potential of acute events to subtly disorganize the hippocampal anatomy. At the molecular level, several events may contribute to this result. Phosphorylation of DCX by cyclin-dependent kinase 5 (Cdk5) reduces its affinity for microtubule binding, enhancing cytoskeletal plasticity, and potentially promoting abnormal migration (Tanaka et al., 2004). In addition, extracellular guidance cues such as reelin, netrins, and semaphorins are critical for granule cell lamination. Disruption of reelin signaling, either through genetic deletion or loss of reelin-secreting interneurons, leads to aberrant migration and positioning of granule neurons (Del Río et al., 1997). Although this event is classically associated with chronic epilepsy, acute severe seizures may be sufficient to transiently disrupt interneuron function and reelin availability, contributing to the altered migration patterns observed in the 25 mg/kg KA condition.

Notably, unlike chronic epilepsy models, we did not observe significant ectopic migration of abGCs into the hilus. This suggests that recurrent seizure activity is required to induce a severe anatomic disorganization coupled to ectopic migration, whereas acute hyperexcitability preferentially affects laminar positioning within the GCL. Soma translocation in hyperexcitable conditions has also been documented. Our observations are in line with such works (Patino and Parent, 2012; Spigelman et al., 1998) as we detected some abGCs with a basal dendrite present.

In addition to positional abnormalities, severe acute seizures associated with a higher proportion abGCs with mature features, consistent with previous evidence that epileptiform activity can accelerate neuronal maturation, particularly by promoting dendritic growth and complexity (Overstreet-Wadiche and Westbrook, 2006). Our findings are consistent with this evidence and suggest that even a single episode of severe hyperexcitability may be sufficient to impact the neurogenic process toward accelerated maturation, as suggested by the increase in Type D abGCs. It is of note that a further fine analysis of abGCs born in 5 mg/kg and 25 mg/kg show that length and number of dendritic branches was not overall increased compared to Sal, which may rely on the fact that for this analysis, we did not specifically chose Type D cells, but analyzed Tom + cells randomly.

Several mechanisms may contribute to the observed accelerated maturation. Depolarizing GABAergic signaling through GABA A receptors promotes functional maturation of abGCs (Ge et al., 2006). In addition, seizure-induced upregulation of BDNF, particularly within dendritic compartments, may facilitate dendritic growth and synaptic integration (Tongiorgi et al., 2004). Together, these findings suggest that acute hyperexcitability can affect the developmental trajectory of abGCs by acting directly upon them or indirectly through changes in the neurogenic niche.

Our results further contribute to the characterization of cells born under hyperexcitable conditions as we now show that cells born under acute seizures also develop pre and postsynaptic structures as mossy fiber boutons, filopodia, and dendritic spines, which provides the features for potential integration into the hippocampal circuit.

### Preservation of global dendritic architecture

At the input level, neither total dendritic length nor the number of dendritic branching points differed significantly between Sal and KA-treated groups at 30 dpl, despite a mild, non-significant trend toward reduction under severe hyperexcitability (25 mg/kg KA-dose). This relative preservation contrasts with the well-documented acute dendritic damage, beading and retraction observed shortly after excitotoxic insults (Swann et al., 2000; Zeng et al., 2007), but is consistent with prior studies showing stabilization and recovery of abGCs dendritic trees architecture during the chronic phase following seizures, when synaptic organization is profoundly altered (Murphy and Danzer, 2011; Walter et al., 2007).

Importantly, this apparent stability must be interpreted in light of the heterogeneity of the abGCs population analyzed. Although severe hyperexcitability increased the proportion of morphologically complex, mature type D neurons, dendritic analyses were performed randomly across all labeled neurons rather than being restricted to a subset. Consequently, subtle increases in dendritic complexity specific to type D neurons may be diluted by the inclusion of less mature abGCs with simpler arbors, particularly given the relatively modest increase in type D cells. Thus, the absence of global changes in dendritic complexity does not exclude cell-type-specific remodeling associated with accelerated neuronal maturation. Overall, these findings suggest strong homeostatic constraints on dendritic growth in abGCs, and further indicate that a single episode of hyperexcitability is not sufficient to induce an overall large-scale reorganization.

### Enhanced dendritic spine density after severe hyperexcitability

In contrast to the stability of dendritic architecture, we observed at the same time point, an increase in dendritic spine density selectively in the severe hyperexcitability group, indicating sustained synaptic remodeling rather than a transient response. Although acute hyperexcitability is associated with spine loss (Isokawa, 1998; Wong and Guo, 2013; Xie et al., 2021) several studies have documented partial recovery or overcompensation of spine density during epileptogenesis, particularly under chronic network hyperactivity (Heysieattalab and Sadeghi, 2021; Isokawa, 2000; Wong and Guo, 2013). Our results align with this literature and support the notion that even a transient hyperexcitability event may drive aberrant excitatory synapse formation or long-term effects on synaptic structures. However, it should also be considered that recurrent discharges could have occurred without being detected by our EEG recordings, thus driving long-term effects on synaptic spines.

Mechanistically, seizure activity alters actin-regulatory pathways involving Rho GTPases, cofilin, and Arp2/3, promoting excessive spine formation or impairing pruning (Chazeau and Giannone, 2016; Newey et al., 2005). In parallel, mTOR pathway activation, strongly implicated in epileptogenesis, promotes protein synthesis and spine growth and has been directly linked to seizure-induced synaptic overgrowth (Zeng et al., 2009). Although this increase in spine density may reflect maladaptive synaptic gain beyond the limits of homeostatic synaptic scaling, it may also indicate accelerated maturation given the enhancement in local input complexity in abGCs, as previously described following high activity (Overstreet-Wadiche and Westbrook, 2006). Further morphological analyses may provide evidence regarding maturation of dendritic spines based on shape and stability (Paulin et al., 2016).

Together, these observations suggest that abGCs retain strong constraints on gross dendritic growth under persistent network dysfunction, and that long-term epileptogenic remodeling may preferentially target synaptic architecture and subcellular specializations rather than inducing large-scale reorganization of dendritic trees. This interpretation aligns with the growing view that chronic hyperexcitability reshapes hippocampal circuits primarily through fine-scale structural and functional plasticity rather than overt alterations in dendritic architecture.

### Differential presynaptic remodeling of mossy fiber boutons across mild and severe hyperexcitability

Mossy fiber bouton (MFB) size strongly correlates with synaptic strength, vesicle pool size, and glutamate release (Nicoll and Schmitz, 2005). The selective enlargement of MFBs following mild acute hyperexcitability (5 mg/kg KA) likely reflects adaptive or compensatory strengthening of presynaptic output, potentially enhancing feedforward excitation onto CA3 targets. Such enlargement reflects enhanced cytoskeletal stabilization and vesicle trafficking, processes regulated by microtubule-associated proteins and activity-dependent BDNF signaling (Danzer et al., 2002; Kapitein and Hoogenraad, 2015). A similar bouton hypertrophy has been reported in models of moderate activity elevation and learning-related plasticity, where increased presynaptic capacity supports circuit adaptation rather than pathology (Danzer et al., 2010; Wiera and Mozrzymas, 2015).

In contrast, the reduction in MFBs size observed at the severe hyperexcitability suggests presynaptic microtubule destabilization, potentially driven by cytoskeletal disruption, impaired axonal transport, or activity-dependent synaptic pruning triggered by excessive calcium influx and metabolic stress (Carletti et al., 2016). This interpretation is consistent with reports showing that severe epileptogenic insults can impair MFB maturation and destabilize presynaptic structures leading to disruption of the axonal and MFB organization despite ongoing axonal sprouting (Buckmaster, 2012; Danzer et al., 2002). Alternatively, it has been shown that MFB reduce their size along maturation (Restivo et al., 2015), being bigger at 4 weeks-old of neuronal age and decreasing at later time points. In this sense, MFBs receiving a strong stimulus in the high hyperexcitability group, could accelerate their maturation and therefore show a reduced area at the time point of our evaluation. However, the increase in filopodia may suggest otherwise.

Despite divergent changes in bouton size, both KA doses induced a significant increase in the number of filopodial extensions from MFBs. Increased mossy fiber filopodial density after hyperexcitability has been linked both to compensatory recruitment of inhibitory interneuron circuits (Pan et al., 2022) and to immature or unstable synaptic remodeling (Danzer et al., 2010), suggesting that its functional significance likely depends on the severity and temporal stage of epileptogenic network reorganization. Under physiological conditions, mossy fiber filopodia are known to preferentially target interneurons, contributing to feedforward inhibition (Acsády et al., 1998; Restivo et al., 2015). Their increase suggests that hyperexcitability alone is sufficient to trigger axonal exploratory growth, independent of dose severity, likely via reactivation of developmental-like growth programs driven by activity-dependent guidance cues and cytoskeletal dynamics (Buckmaster, 2012; Galimberti et al., 2006). This finding aligns with classical observations of mossy fiber sprouting and excessive filopodial growth in temporal lobe epilepsy models (Buckmaster and Dudek, 1997; Sutula et al., 1989). Importantly, increased filopodial number in the absence of coordinated bouton enlargement (as in the severe hyperexcitability group) may reflect structurally immature or unstable synaptic contacts, further contributing to circuit dysregulation (Danzer et al., 2010).

Collectively, our data support a model in which moderate hyperexcitability (5 mg/kg KA) is associated with increased presynaptic bouton size and exploratory connectivity, whereas severe hyperexcitability (25 mg/kg KA) is associated with increased postsynaptic spine density and reduced presynaptic bouton size.

Importantly, these changes occur without overt alterations in dendritic architecture, underscoring the fundamentally synaptic and subcellular nature of epileptogenic plasticity.

### Relationship between hyperexcitability, neurogenesis, and hippocampal-dependent memory performance

At the behavioral level, both KA doses were associated with impaired object location memory (OLM), indicating that acute hyperexcitability disrupts hippocampal-dependent spatial processing. Part of this deficit may be related to alterations in neurogenesis, as previous studies have shown that aberrant neurogenesis can be linked to cognitive impairments in epilepsy (Cho and Hsieh, 2024). However, the OLM task also relies on coordinated activity across multiple hippocampal subregions, including CA1 and CA3 (Benkovic et al., 2004), suggesting that a hyperexcitability event, regardless of its intensity is sufficient to alter the function of the hippocampal circuit, which reflects in spatial memory deficits (Fernandes et al., 2025). In contextual fear conditioning (CFC), impairments were most evident during the acquisition phase, particularly in the 5 mg/kg KA group. This pattern is consistent with transient disruptions in hippocampal circuit function during learning, followed by compensatory mechanisms that support memory expression at later stages. Supporting this idea, prior work from our laboratory demonstrated that a focal KA-induced dentate gyrus lesion initially impairs contextual memory but recovers by 30 days post lesion (Hernández-Mercado and Zepeda, 2022), coincident with increased functional recruitment of mature abGCs (Aguilar-Arredondo and Zepeda, 2018).

### Functional implications of seizure-induced abGCs

The functional contribution of seizure-induced neurogenesis remains highly debated. On the one hand, abGCs generated under chronic and severe hyperexcitable conditions have been proposed to promote epileptogenesis by forming recurrent aberrant local circuits and synchronizing with CA3 pyramidal neurons (Scharfman et al., 2000; Shapiro and Ribak, 2006). Consistent with this view, ablation of seizure-generated neurons reduces seizure frequency, supporting a pro-epileptogenic role (Jung et al., 2006).

On the other hand, evidence also supports a compensatory or protective role for abGCs. In certain contexts, seizure-induced neurons exhibit reduced excitatory input and enhanced inhibitory innervation, resulting in lower intrinsic excitability compared to neurons generated under physiological conditions (Jakubs et al., 2006).

In the present study, the absence of recurrent seizures suggests that abGCs generated under acute hyperexcitability are unlikely to drive epileptiform activity at the analyzed time point. However, their atypical positioning, altered maturation, abnormal growth of MFBs and multiple but subtle alterations, may reshape hippocampal circuitry, influencing information processing and behavioral performance.

Taken together, our findings indicate that acute hyperexcitability differentially shapes adult hippocampal neurogenesis depending on seizure severity, by altering neuronal positioning and maturation features rather than overall neuron number. These changes are associated with deficits in spatial memory performance, rather than in overall hippocampal-mediated memory. Therefore, considering the precise contribution of hyperexcitability-driven alterations in each hippocampal synaptic relay may provide further insights into specific functional deficits. Future studies integrating functional analyses, connectivity, and electrophysiological characterization of abGCs will be essential to delineate how acute seizure-induced remodeling of adult neurogenesis contributes to circuit reorganization and cognitive outcomes.

## Data Availability

The raw data supporting the conclusions of this article will be made available by the authors, without undue reservation.
