# Supplementary figures and images for: Acute hyperexcitability differentially affects hippocampal neurogenesis features and spatial memory
Source: Front Cell Neurosci. 2026 Jun 2;20:1833859. doi: 10.3389/fncel.2026.1833859 (PMC13268882; doi:10.3389/fncel.2026.1833859)

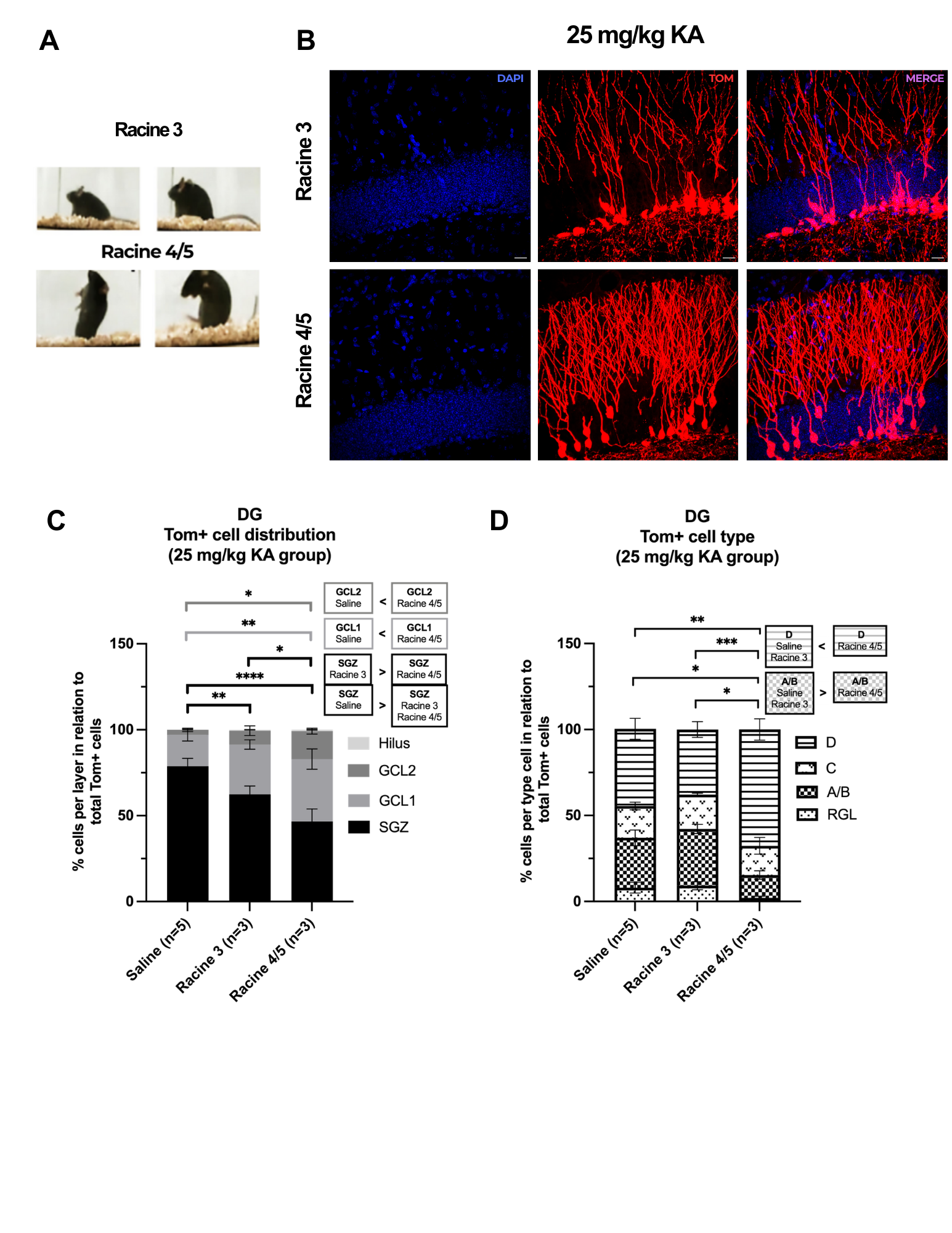

Supplement: Supplementary file 1 [file Image_1.PNG]

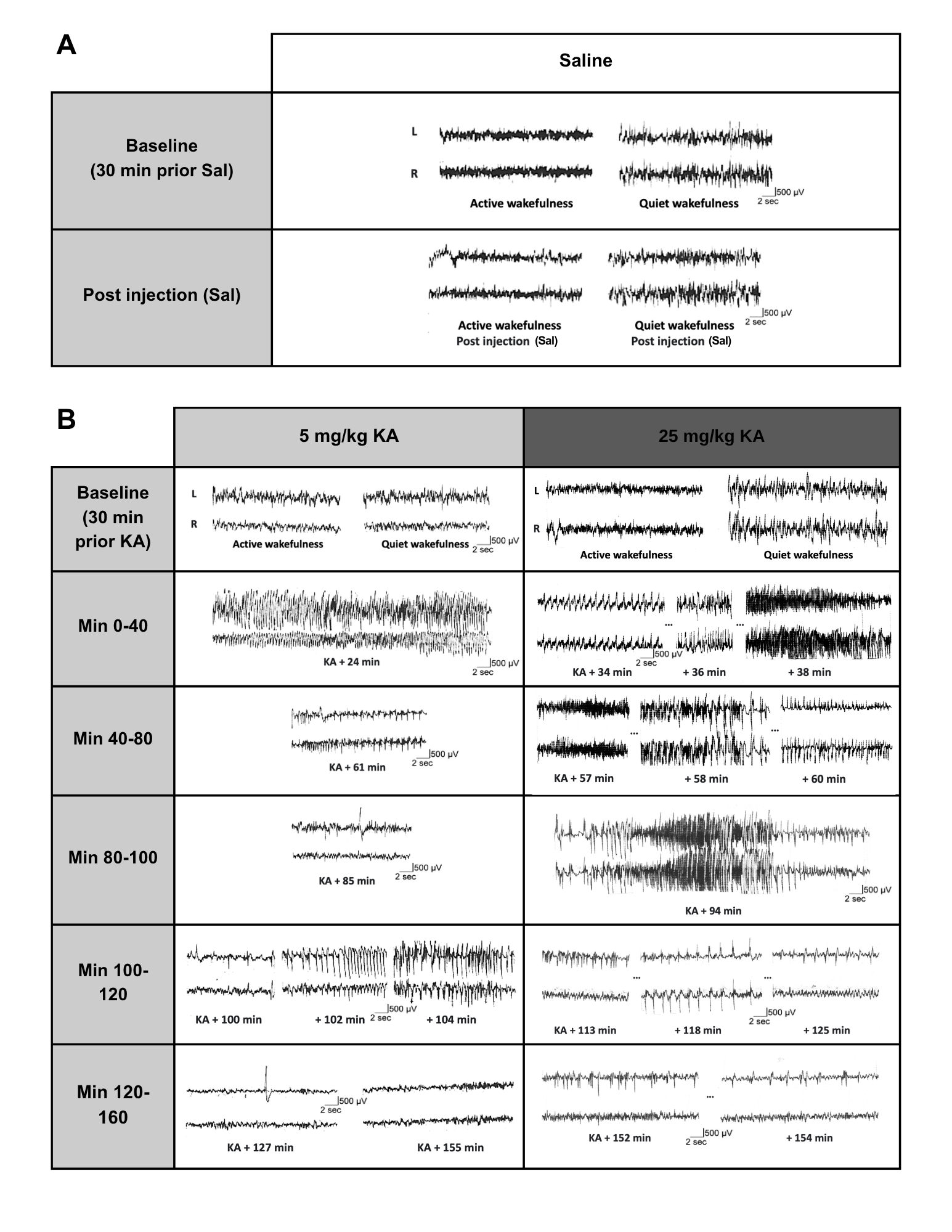

Supplement: Supplementary file 2 [file Image_2.PNG]

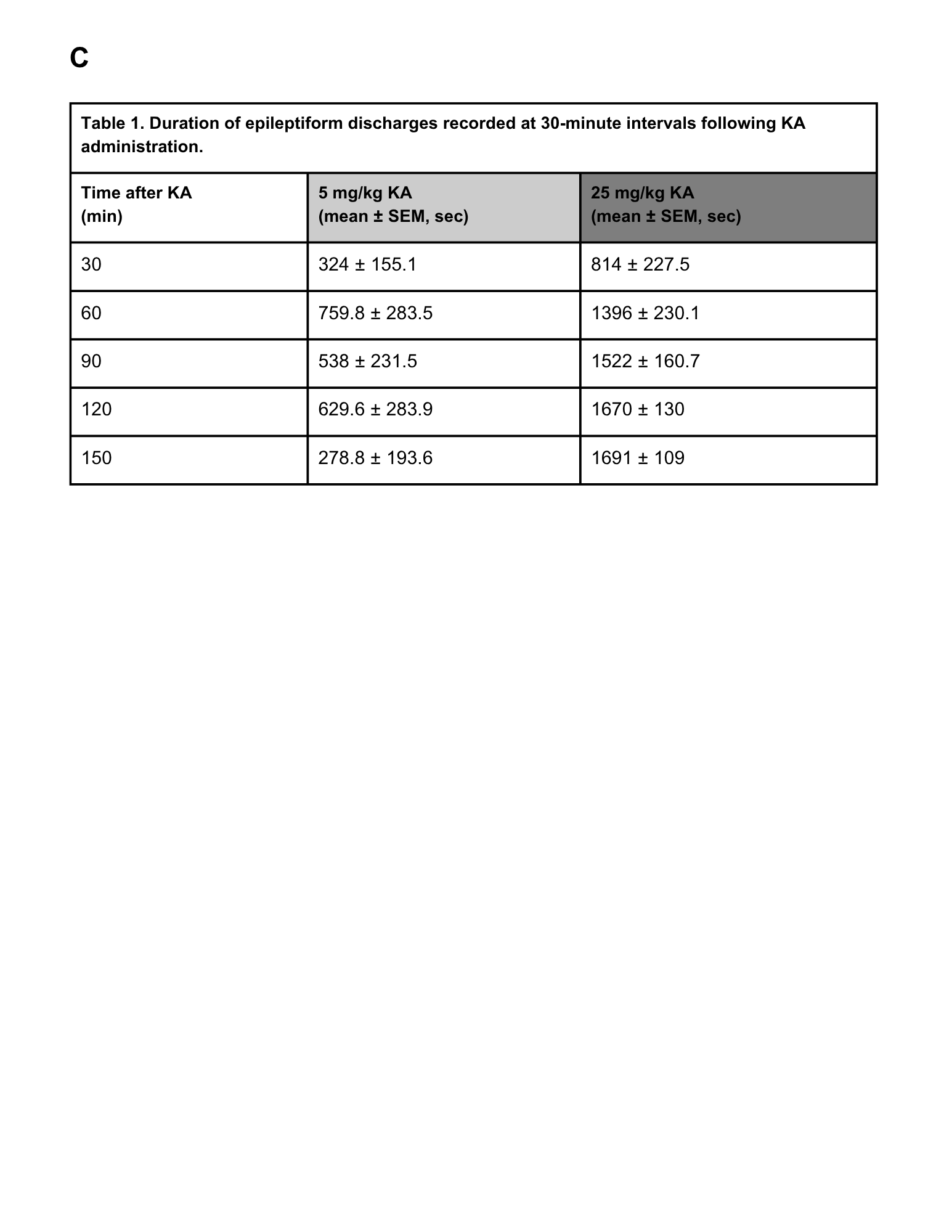

Supplement: Supplementary file 3 [file Image_3.PNG]
